# Transcriptome free energy can serve as a dynamic patient-specific biomarker in acute myeloid leukemia

**DOI:** 10.1038/s41540-024-00352-6

**Published:** 2024-03-25

**Authors:** Lisa Uechi, Swetha Vasudevan, Daniela Vilenski, Sergio Branciamore, David Frankhouser, Denis O’Meally, Soheil Meshinchi, Guido Marcucci, Ya-Huei Kuo, Russell Rockne, Nataly Kravchenko-Balasha

**Affiliations:** 1 Division of Mathematical Oncology and Computational Systems Biology, Department of Computational and Quantitative Medicine, Beckman Research Institute, City of Hope National Medical Center, Duarte, CA 91010 USA; 2 The Institute of Biomedical and Oral Research, Faculty of Dental Medicine, The Hebrew University of Jerusalem, P.O.B. 12272, Ein Kerem, Jerusalem, 91120 Israel; 3 Department of Diabetes and Cancer Discovery Science, Arthur Riggs Diabetes and Metabolism Research Institute, Beckman Research Institute, City of Hope National Medical Center, Duarte, CA 91010 USA; 4 Clinical Research Division, Fred Hutchinson Cancer Center, 1100 Fairview Ave N, D5-112, Seattle, WA 98109 USA; 5 Department of Hematological Malignancies Translational Science, Gehr Family Center for Leukemia Research, Beckman Research Institute, City of Hope National Medical Center, Duarte, CA USA

**Keywords:** Biophysics, Cancer, Systems biology

## Abstract

Acute myeloid leukemia (AML) is prevalent in both adult and pediatric patients. Despite advances in patient categorization, the heterogeneity of AML remains a challenge. Recent studies have explored the use of gene expression data to enhance AML diagnosis and prognosis, however, alternative approaches rooted in physics and chemistry may provide another level of insight into AML transformation. Utilizing publicly available databases, we analyze 884 human and mouse blood and bone marrow samples. We employ a personalized medicine strategy, combining state-transition theory and surprisal analysis, to assess the RNA transcriptome of individual patients. The transcriptome is transformed into physical parameters that represent each sample’s steady state and the free energy change (FEC) from that steady state, which is the state with the lowest free energy.

We found the transcriptome steady state was invariant across normal and AML samples. FEC, representing active molecular processes, varied significantly between samples and was used to create patient-specific barcodes to characterize the biology of the disease. We discovered that AML samples that were in a transition state had the highest FEC. This disease state may be characterized as the most unstable and hence the most therapeutically targetable since a change in free energy is a thermodynamic requirement for disease progression. We also found that distinct sets of ongoing processes may be at the root of otherwise similar clinical phenotypes, implying that our integrated analysis of transcriptome profiles may facilitate a personalized medicine approach to cure AML and restore a steady state in each patient.

## Introduction

Acute myeloid leukemia (AML) is an aggressive hematopoietic malignancy with a poor overall survival rate. This is a highly heterogenous disease driven by combinations of genomic mutations, epigenetic alterations, and biochemical signaling processes which result in highly variable disease progression, treatment response, and outcomes among individual patients. The genetic heterogeneity underlying AML and outcome disparities call for new approaches for individualized clinical assessment and treatment selection. In recent years, the transcriptome has emerged as a promising avenue for identifying prognostic markers in AML.

Several recent studies have demonstrated the utility of transcriptome signatures in AML which refine disease classification, provide risk stratification, and predict prognosis. Transcriptome profiling has aided in the identification of distinct molecular subgroups within AML, enhancing disease classification beyond traditional morphological and cytogenetic criteria which are crucial for determining appropriate treatment strategies. For example, Papaemmanuyouil et al.^1^ utilized RNA-seq data to refine the World Health Organization (WHO) classification system for AML, resulting in improved disease classification and prognostic stratification. Similar studies have used RNA-seq data to develop gene expression-based scores to predict treatment response, progression-free and overall survival, and define minimum residual disease in AML^2–4^. However, approaches that rely on differential expression or correlation analysis lack an underlying theoretical model of the disease to inform the interpretation of all ongoing processes in each individual which is crucial for personalized diagnostics and treatment selection. To address this gap in methodology, we integrated two approaches from physics and chemistry to create a patient-specific biomarker that can be used to identify the individualized state of the disease that can be used in personalized diagnostics and individualized treatment in the future.

**Fig. 1:**
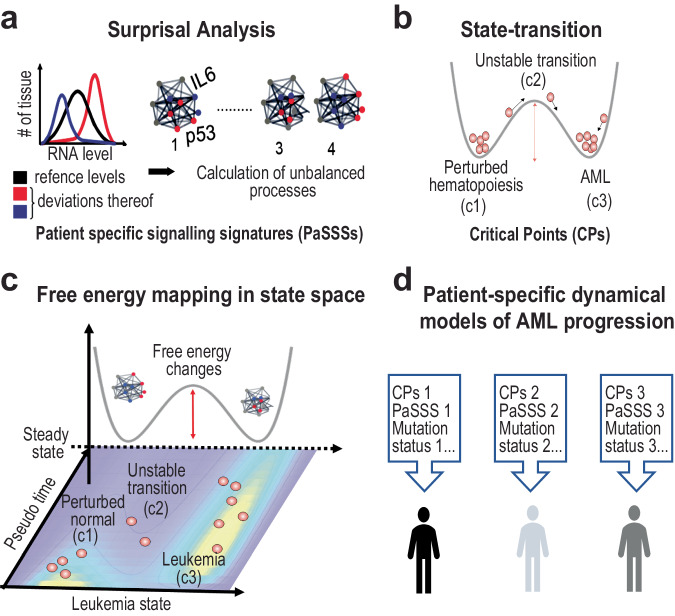
Characterizing AML stages by combining the state-transition approach with SA-based PaSSS analysis. **a** Surprisal analysis, PaSSS calculation and (**b**) state-transition model are carried out as described in Methods. **c** Schematic representation of mapping free energy within the state-transition state-space and (**d**) construction of patient-specific dynamical models of AML progression.

The first method is surprisal analysis (SA), which is an analytical approach rooted in information theory that is used to study complex systems in physics and biology, including diseases such as AML. SA utilizes principles from physics, chemistry, and thermodynamics, to model the system as a collection of information-carrying entities (mRNA molecules) that respond to constraints imposed by perturbations to the system. Any environmental or genetic perturbation is viewed as a constraint that prevents the system from reaching its most stable, steady state, which is associated with the lowest free energy state of the system^5,6^. SA identifies both states in each sample: the steady state and the constrained state, where specific RNA patterns are most active. In the context of cancer, SA focuses on the coordinated expression of mRNA molecules involved in regulation in AML-related perturbed hematopoiesis (Fig. 1a). By analyzing the patterns in gene expression profiles, SA helps uncover the underlying dynamics and regulatory mechanisms of the observed AML phenotype.

We have reported in several prior studies that the baseline transcriptional state (steady state) is common and invariant between normal and diseased tissues^6^. This observation allows us to identify deviations from the steady state as constraints on the system that imply *unbalanced or ongoing* biological processes associated with disease or normal states. Each patient may harbor a unique set of unbalanced processes, which we refer to as patient-specific signaling signatures (PaSSS). PaSSSs may be used to refine disease classification, quantify biological heterogeneity^7^ or identify therapeutic strategies aimed at modifying the unbalanced processes to restore the system to the baseline steady state. Here, we extend the concept of PaSSS to include individualized change in free energy (FEC) to reduce the transcriptome to a single physical parameter which quantifies the deviation from the baseline steady state of the system to create a patient-specific biomarker of the disease state.

The second method we integrated with SA was a mathematical model based on state-transition theory, which has a rich history of applications to epithelial to mesenchymal transitions (EMT) and origins in Waddington’s famous epigenetic landscape^8^. Because AML is a dynamic, evolving disease, we applied our recently published mathematical model of AML progression to inform the interpretation of PaSSSs and FEC. The model applies the concept of phase transition in thermodynamics to AML disease evolution. From this physics-based perspective, AML initiation and progression are modeled as a state transition of the transcriptome, where the transcriptome is represented as a particle undergoing Brownian motion in a potential energy landscape^9^. The potential is composed of three states, which are a healthy state, an unstable transition state, and an AML state (Fig. 1b). In a state of normal healthy hematopoiesis, the transcriptome particle moves in a potential with a high energy barrier that reduces the probability to transition from a healthy state to an AML state. In this model, leukemogenic events such as mutations and chromosomal abnormalities act to reduce the energy barrier of the potential, and as a result, increase the probability of transition from a healthy state to an AML state. We have previously shown that the state-transition model can be used to track changes in the transcriptome over time and identify critical points which we can accurately predict disease progression and treatment response in a mouse model of AML^9,10^.

Here we combine the state-transition model with surprisal analysis to analyze free energy changes that occur at state-transition critical points that predict AML progression (Fig. 1c). We postulated that mapping FEC into an AML state-space could provide a high resolution, patient-specific characterization of state-transition critical points and via biological interpretation of PaSSSs. We show how each sample can be characterized in terms of how many ongoing processes every patient has, and how the patient population can be accurately classified (Fig. 1d). Using publicly available RNA-seq datasets for hundreds of AML patients^11–15^, we show that the transcriptomes from peripheral blood (PB) and bone marrow (BM) AML samples have higher free energy states than normal controls, and thus they are less stable from a thermodynamic perspective. Because a change in free energy is required for disease progression, we propose that identifying key molecular pathways responsible for deviations from the steady state could aid in the patient-specific diagnosis and identification of therapy targets that would restore the steady state in the tissue. We observed that AML samples with comparable FEC levels or clinical characteristics could be defined by different barcodes, or sets of unbalanced processes, implying that this information might be used to determine tailored therapies in subgroups of individuals with similar clinical or thermodynamic characteristics.

## Results

### **Surprisal analysis reveals an invariant transcriptome steady state**

**Fig. 2:**
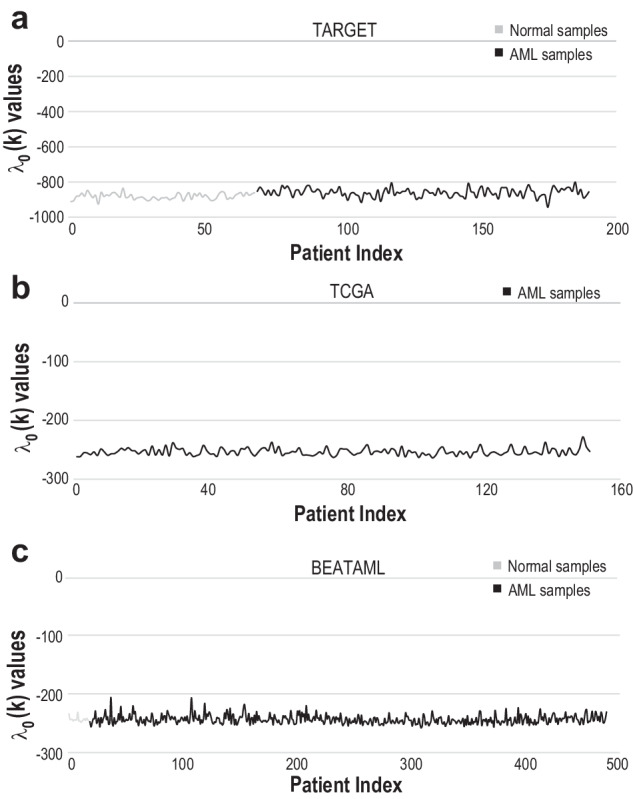
Transcriptome steady state is invariant to normal and leukemic samples. Amplitudes of steady state ${\lambda }_{0}(k)$ for normal (light gray) BM and PB samples (dark gray and black respectively) of TARGET (**a**), TCGA (**b**) and BEATAML (**c**) datasets.

By utilizing 858 AML samples from three publicly available datasets (Table 1), we quantified each sample’s steady state, which is equivalent to the minimal free energy using surprisal analysis and transcriptome measurements (Methods and Fig. 2). The steady state has a large and unchanging amplitude ${\lambda }_{0}\left(k\right)$ over all normal and AML samples. This result implies that the transcriptome steady state is an *invariant* state and remains stable across disease states and even during transition from a normal to an AML state. Moreover, the steady state is the most significant contributor to the overall transcriptome profile, as its amplitude is significantly higher in comparison with the amplitudes of the other unbalanced processes (${\lambda }_{0}\left(k\right) \,>\, {\lambda }_{\alpha }\left(k\right)$ for $a >\, 0$ and all $k$). These processes deviate from the steady state (Supplementary Fig. 1a-c, Supplementary Table 1) and constitute groups of co-expressed transcripts (Supplementary Table 2). In every dataset, at least 12 unbalanced processes that efficiently reproduced the experimental data were found (Methods, Supplementary Fig. 2), indicating that each dataset has at least 12 dimensions.

**Table 1:** Summary of data used in this study

Data set	Age range (years)	Tissue sample type	Number of samples	Mutation (WT1, NPM1, CEBPA)	Primary fusion
TARGET	0.3 - 28.4	Bone marrow	105	WT1: 3	CBFB-MYH11: 105
		Peripheral blood	21	WT1: 2	CBFB-MYH11: 21
		Normal bone marrow	84		
BEATAML	2.04 - 87.2	Primary blood-derived cancer, bone marrow	139	NPM1: 42, CEBPA: 9	CBFB/MYH11: 10
		Primary blood-derived cancer, peripheral blood	89	NPM1: 21, CEBPA: 6	CBFB/MYH11: 12
		Recurrent blood-derived cancer, bone marrow	106	NPM1: 16, CEBPA: 11	CBFB/MYH11: 1
		Recurrent blood-derived cancer, peripheral blood	142	NPM1: 34, CEBPA: 4	CBFB/MYH11: 4
		Blood derived normal	21		
TCGA	21.6 - 88.6	Primary blood-derived cancer, peripheral blood	151		
GSE133642	0.1-1	Peripheral blood-derived cancer	12		Cbfb-MYH11: 12
		Blood derived normal	14		
Total			**884**

Gene Ontology analysis, used to interpret the unbalanced processes biologically, revealed that interleukin pathways such as IL1, IL2, IL8 and IL10, MAPK, NFkB^16–27^ and migration-related pathways, which are known to be involved in AML progression, were associated with multiple unbalanced processes characterizing AML across all three datasets (Supplementary Note 1). For example, the IL1 pathway was found in 3 unbalanced processes characterizing the TARGET dataset and in four processes characterizing TCGA and BEATAML. IL2-related categories were found among induced transcripts in 4 unbalanced processes characterizing the TARGET dataset, in 6 characterizing TCGA and in 4 processes characterizing BEATAML (Supplementary Tables 3–5).

### PaSSSs refine AML disease classification

Despite common and known pathways and processes being present in all datasets, we found that not every patient developed every process. The number of patients with unbalanced processes with lower indices (*α* = 1,2,..) was higher than the number of patients with higher indices (such as *α* = 6,7.., Supplementary Fig. 1). Furthermore, these appeared in different combinations in individual patients, suggesting a personalized approach method of AML disease classification based on PaSSS (Methods) which is independent of, but complementary to, cytogenetics or mutations. Specifically, we found that PaSSS of *each* AML sample could be characterized by a patient-specific set of approximately 1-3 unbalanced processes out of *n* = 12–18 found in the datasets. AML subtypes with 40 different PaSSSs were discovered to be recurrent in the TARGET, 141 PaSSSs in BEATAML, and 80 in TCGA datasets (Supplementary Tables 6, 7). This result is consistent with our PaSSS-based analysis of other cancer types^7,28^ in which a certain cancer type (e.g. breast, melanoma) could be sub-classified into dozens of different PaSSS-based subtypes representing different patients.

### Free energy changes are associated with state-transition critical points

Using the PaSSS categorization, we could clearly identify a relatively high heterogeneity within each disease stage (Supplementary Table 7), suggesting that discriminating between distinct transition points may be difficult. We proposed computing a single, quantitative value that indicates a whole change in each sample’s transcriptome state. We anticipated that such a measure may more reliably differentiate between various AML stages, yielding a diagnostic value. To this end, we calculated a change in free energy in each AML sample across all datasets and mapped these values into the AML state space.

A free energy change depicts the full, personalized molecular (transcriptomic in this case) change since it is based on a patient-specific set of unbalanced processes found in each sample. The sum of these processes, including their amplitudes, represents FEC as a whole from the steady state, as we sum up all deviations from the steady state due to patient-specific unbalanced processes (Methods). Consequently, FEC is an integrated value that incorporates the identified dimensions, hence reducing the multitude of distinct data dimensions to a single, personalized, informative value. Equation 8 computes FEC in dimensionless units (Methods and refs. ^29,30^). To convert FEC to thermodynamic term we can multiply it by *RT*.

**Fig. 3:**
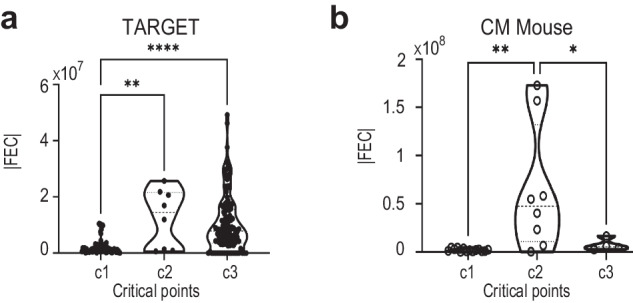
Transcriptome free energy changes are associated with state-transition critical points. **a** For the TARGET dataset, we observed higher mean FEC (computed as absolute values) at the unstable transition point (${c}_{2}$) with significant differences in FEC between ${c}_{1}$ and ${c}_{2}$ (*p* = 0.004), ${c}_{1}$ and ${c}_{3}$ (*p* < 0.001) by ANOVA. The difference in FEC between ${c}_{2}$ and ${c}_{3}$ was not statistically significant. **b** For the CM mouse data (GSE133642)^9^, we observed higher mean FEC at the unstable transition point (${c}_{2}$) with significant differences in in FEC between ${c}_{1}$ and ${c}_{2}$ (p = 0.002) and ${c}_{2}$ and ${c}_{3}$ (p = 0.045) by ANOVA.

We observed significant differences in FEC between the state-transition critical points, with higher FEC at the unstable transition point (${c}_{2}$) as compared to the normal state (${c}_{1}$) (Fig. 3a, Supplementary Table 8). This result was confirmed using longitudinal data collected from an inducible mouse model of AML (GSE133642)^9^ (Fig. 3b). Interestingly, a reduction in average FEC towards ${c}_{3}$ was detected in the AML mouse model and in some samples in the BEATML dataset (Supplementary Fig. 3), suggesting that FEC levels at ${c}_{3}$ can be age dependent (TARGET is a pediatric dataset, whereas BEATMAL has samples up to the age of 87, and the mouse model contains samples from mice aged 1 to 12 months). A tendency toward decreasing FEC levels at the more advanced state (${c}_{3}$) (Fig. 3 and Supplementary Fig. 3) suggests a process of state stabilization at more advanced states of the disease in some cases. Additionally, we discover a strong relationship between the FEC values and the number of processes per sample (Supplementary Fig. 4). These results suggest that FEC may be used as a transcriptome-based diagnostic tool to distinguish between disease stages without regard to morphology or cytogenetics. Additional characterization of AML states can be found in Supplementary Note 1 and Supplementary Fig. 5.

### Similar clinical phenotypes can result from different PaSSSs

The study’s key finding is that the FEC simultaneously provides two significant and patient-specific characterizations: first—a diagnostic parameter for stage classification (when it is represented by a single FEC value); second–a full molecular characterization when FEC is broken down back into the unbalanced processes comprising each PaSSS. Making treatment decisions requires molecular characterization of personalized networks^7,31^. Thus, once a stage of a sample is identified it should be decomposed back for full characterization.

We find that each AML state can be split into many PaSSS-driven subtypes, pointing to patient heterogeneity within each disease state (Supplementary Table 7). Moreover, when we look for a possible link between PaSSSs, recognized AML biomarkers, and clinical characteristics, the picture becomes more complicated.

**Fig. 4:**
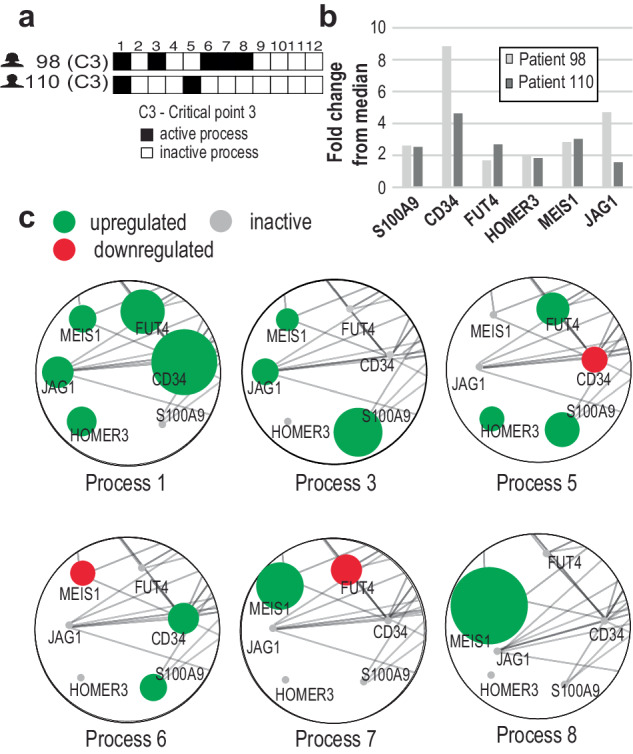
Similar gene expression levels in different patients may be attributed to different unbalanced processes. **a** Transcriptome-barcodes of two AML patients from the TARGET dataset (bone marrow samples), representing the patient-specific combination of unbalanced processes. These patients were classified as being in ${c}_{3}$ critical point by ST analysis. **b** The fold changes of six AML biomarkers (see supplementary methods) S100A9, CD34, FUT4, HOMER3, MEIS1, and JAG1 were up-regulated in both patients relative to their median expression levels across 190 other patients in the TARGET data set. **c** Detail of a network diagram of unbalanced processes, comprising the barcodes of patients 98 and 110 (**a**) and in which the selected biomarkers whose levels are most influenced by those processes. Green denotes up-regulation, red denotes down-regulation, and gray denotes no change. The size of the biomarker indicates its relative weight in each process. Functional connections are derived from the STRING database.

For example, PaSSS of patient 98 (PANWHP) is characterized by a combination of processes 1, 3, 6-8, whereas patient 110 (PAWZUZ) harbors a combination of processes 1 and 5 (Fig. 4a). Figure 4b, c illustrates six well-known AML biomarkers (Supplementary Note 2) that are *all induced* in these patients. However, their induction is linked to different unbalanced processes. For example, CD34 induction (Fig. 4b) is associated with unbalanced process 1 in both cases (Fig. 4c). Yet, another process contributes to the high levels of CD34 expression in patient 98. In this case, CD34 induction was attributed to process 6 in addition to process 1 (C. 4b, c). Similar examples from BEATAML and TCGA datasets can be found in SI (Supplementary Figs. 6, 7). This suggests that using biomarkers to characterize tumors only results in a partial characterization.

**Fig. 5:**
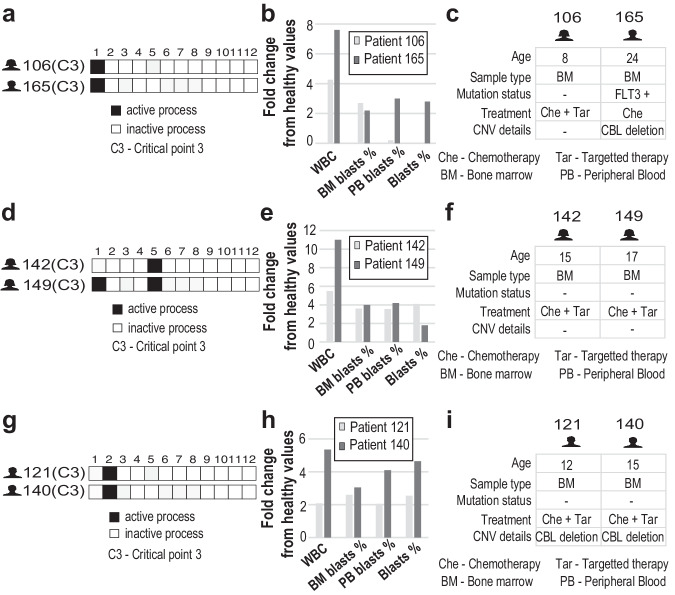
Comparison of barcodes and clinical features. **a** Two patients (TARGET), having the same PaSSS barcodes, are shown. **b** Fold change of different clinical markers are shown relative to their healthy values. **c** Clinical data of these two patients are shown, demonstrating that patients with the same set of unbalanced processes can have different clinical data. Pathology assessment of cell morphology was used to determine the percent of PB (peripheral blood) and BM (bone marrow) blasts. Leukemic blast percentage (Blasts %) was also quantified using clinical flow cytometry. **d** Two patients, having the different sets of unbalanced process are shown. **e** Fold change of different clinical markers are shown relative to their healthy values. **f** Clinical data of these two patients are shown, demonstrating that patients with different sets of unbalanced processes can have similar clinical data. **g** Two patients, having the same barcodes are shown. **h** Fold change of different clinical markers are shown relative to their healthy values. **i** The same clinical data of these two patients are shown, demonstrating that in some (rare) cases we find patients with same barcodes and the same clinical data.

We find also that patients with similar clinical phenotypes may have different PaSSS and vice versa - similar PaSSSs may be associated with different clinical phenotypes (Fig. 5). For example, two patients with the *same* PaSSS harboring process 1 (Fig. 5a, patient 106 PARJRG, female, 8 years and 165 PAXDDY, male, 24 years) had different clinical characteristics. In addition to the sex and age differences, the leukemic blast percentages in the peripheral blood were highly discordant (Fig. 5b). Patient 165 (PAXDDY) was also found to have the FLT3 mutation which occurs in about one-third of the newly diagnosed AML cases, was treated with chemotherapy, and additionally harbored a CBL deletion. Patient 106 (PARJRG) did not have any mutations and was treated with chemotherapy and targeted therapy (Gemtuzumab, Ozogamicin, and Mylotarg, Fig. 5c).

PAWTHU (patient 142) and PAVDBT (patient 149) are examples of patients with similar clinical phenotypes but different PaSSS (Fig. 5d–f). Patients PAWTHU and PAVDBT were females of similar age, with similar WBC and blast levels in BM and PB and were also treated in a similar manner with chemotherapy and bortezomib. However, they had different PaSSSs (Fig. 5d), showing that the patients with very close clinical data can have different tumor biology. The distributions of patient pairings with comparable clinical phenotypes but distinct PaSSSs, and vice versa, are shown in Supplementary Figs. 8–10. Interestingly, patients with distinct PaSSSs but comparable clinical features (5-6 similar features out of examined 9 in TARGET and TCGA, and 1-2 out of 3 in BEATAML) form the greatest number of potential pairs (Supplementary Figs. 8–10). This suggests that identical clinical characteristics might not be sufficient to appropriately select the best course of treatment and that PaSSS and FEC characterization should be added to provide a more precise patient characterization.

In some cases, we found patients with similar PaSSSs and similar clinical characteristics (Fig. 5g–i). For example, two male patients, PAXKES (patient 121) and PAWDBB (patient 140), were from the same age group, had the same PaSSSs, and similar WBC and blast levels. They received similar treatment (chemotherapy and Bortezomib) and both had CBL gene deletion. Similar examples from BEATAML and TCGA datasets can be found in SI (Supplementary Figs. 11, 12).

## **Discussion**

We combined two approaches from physics and chemistry to analyze 884 RNA transcriptomes derived from peripheral blood and bone marrow from normal and AML patients with different mutations, cytogenetics, ages, and disease stages from three independent datasets and a mouse model to investigate how PaSSSs and free energy variations might contribute to the patient-specific AML characterization.

First, we observe that the steady state is *invariant across all analyzed AML samples*, meaning that all—normal and diseased samples share similar transcriptome steady states. Based on this result, we postulate that there may be a steady-state transcriptome “core” that is essential and compatible with life.

Next, we demonstrate that, when FEC is broken down into the unbalanced processes that make up each PaSSS, it simultaneously provides two important and patient-specific characterizations: first, a diagnostic parameter for stage classification (when it is represented by a single FEC value); and second, a molecular characterization of the network alterations. We have shown earlier for other cancer types that PaSSS dictates personalized combination of drug treatments^7,31^. Here, we expand on this idea by demonstrating that each PaSSS may be utilized for diagnostic stage characterization in addition to personalized molecular characterization after it is integrated into FEC.

We found that AML tissues have higher FEC in comparison with normal tissues. Mapping free energy changes to critical points, as estimated from the AML state space, provided additional information about the disease state. Distributions of FEC by critical points showed the highest mean and variance in free energy changes in the unstable transition state (${c}_{2}$). The results were validated with data collected from a mouse model of AML which followed disease progression longitudinally over time. This suggests that patients whose disease is in the ${c}_{2}$ state have a higher probability to undergo state transition either to normal hematopoiesis (remission to ${c}_{1}$) or progression state $({c}_{3})$ as found for at least several cases in BEATAML or mouse model. This suggests that a “${c}_{2}$” transcriptome-based diagnosis may have a better chance for an effective, patient-specific treatment, as their less stable disease state would be easier to modify. Although FEC decreases in some ${c}_{3}$ samples, in many cases it remains higher than in the ${c}_{1}$ state. This finding implies that although the progressive stage of the disease becomes more “stable” it still can be treatable in at least certain cases and that FEC may serve as a predictive clinical parameter.

The dynamical model combined with FEC may also enable early detection of potential malignant transformation as well as disease progression and response to therapy. The notion within cancer researchers that transformed cells are more sensitive to the suppression of overexpressed or hyperactivated signaling proteins than normal cells^32,33^ gives support to this claim. Since FEC calculations directly account for overexpression of molecular pathways, FEC provides a thermodynamic explanation for the transformed/cancer cell’s increased sensitivity to drugs. Furthermore, we show that this sensitivity increases significantly when the drug combination is dictated by PaSSS^7,31^. The use of dimension reduction methods such as the SVD to identify energy landscapes and transition states of dynamical systems has been applied to metabolism EMT networks, and normal to cancer epigenetic transitions^8,34,35^, however, to our knowledge, no prior approaches have used personalized SA (PaSSS) to interrogate biological processes associated with cancer transition states.

An important result of this analysis is the high degree of heterogeneity characterizing all AML datasets. We found that each AML sample had a combination of ~ 3 active unbalanced processes on average. We found 40 different PaSSS combinations (barcodes) in 122 TARGET samples, 141 combinations in 469 BEATAML, and 80 in 151 TCGA samples. We also found that although certain patients expressed similar AML biomarkers, they harbored *different* PaSSSs. We observed the same phenomena when we looked at the clinical parameters of different samples. We found that AML patients could have similar clinical and cytogenetics and yet harbor different combinations of unbalanced processes, whereas samples with different clinical parameters could have the same combination of unbalanced process.

We did not observe a generalizable correlation between FEC and overall survival in any of the datasets. This is likely due to several factors known to limit inference from database studies, including selection bias and technical variations in data acquisition. Additional prospective studies are required to test and validate FEC as a prognostic marker and PaSSSs as a potential disease classification metric.

Our findings emphasize the complexity of AML and show that clinical, morphological, and cytogenetic features may benefit from the addition of PaSSS-based subtyping to provide an additional degree of biological resolution that can be used to enhance diagnosis and identify therapies tailored to individual patient disease characteristics in the future^7,31^.

## Methods

### Datasets

A total of 884 samples from three datasets were used in this study (Table 1). The Therapeutically Applicable Research to Generate Effective Treatments (TARGET) dataset^11^ is a pediatric study, from which we analyzed 84 normal samples and 126 AML inv(16) samples^12^, consisting of 105 bone marrow and 21 peripheral blood samples with ages from 0.3 to 28 years. AML samples with the inv(16) karyotype were selected from the TARGET study based on our previous experience with a mouse model of inv(16) AML. TARGET data was processed as described in Huang et al.^12^. The average values of the samples were taken for patients with duplicate/multiple samples for SVD analysis, resulting in 122 distinct AML samples. Two additional datasets from the BEATAML study^15^ and the Cancer Genome Atlas (TCGA)^13^, were downloaded from the Genomic Data Commons (GDC) portal^14^ using GDCRNATools^36^ (data query included in supplemental data). The BEATAML dataset included 21 normal and 476 AML samples consisting of 231 peripheral blood and 245 bone marrow samples with ages 2 to 87, and TCGA 151 AML peripheral blood samples, with ages 21 to 88 years. Normal blood or bone marrow samples corresponding to AML patients were not found in the TCGA dataset, limiting the analysis of these samples. Gene counts were normalized based on TPM for TARGET, and FPMK for BEATAML and TCGA. All clinical data are listed in Supplementary Table 9. Time-series peripheral blood samples of an inducible mouse model of AML were used to validate results (GSE133642)^9^. The data from the mouse model included 14 normal samples and 12 AML samples.

### State-transition model

The AML state-transition model represents the transcriptome as a particle undergoing Brownian motion in a double-well quasi-potential landscape characterized by critical points ${c}_{1}$, ${c}_{2}$, and ${c}_{3}$ and scaling coefficient $\alpha$ as

1 $$\nabla {U}_{p}=\alpha\left(x-{c}_{1}\right)\left(x-{c}_{2}\right)\left(x-{c}_{3}\right)$$

The term quasi-potential is used to indicate the concept of a potential energy, but without physical units, and is subsequently referred to simply as a potential. The position of the transcriptome particle over time is denoted ${X}_{t}$, and is given by Langevin equation of motion as

2 $$d{X}_{t}=-\nabla {U}_{p}({X}_{t}){dt}+\sqrt{2{\beta }^{-1}}d{B}_{t}$$

where ${B}_{t}$ is a Brownian stochastic process that is uncorrelated in time $\langle {B}_{i},{B}_{j}\rangle ={\delta }_{i,j}$ with diffusion coefficient ${\beta }^{-1}$. The probability distribution, $P(x,t)$, for a transcriptome particle at location $x$ and time $t$ is given by the solution to the Fokker-Planck equation

3 $$\frac{\partial }{\partial t}P\left(x,t\right)=-\frac{\partial }{\partial x}\left(\nabla {U}_{p}\left(x\right)P\left(x,t\right)\right)+\frac{{\partial }^{2}}{\partial {x}^{2}}\left({\beta }^{-1}P\left(x,t\right)\right)$$

The probability distribution is used to predict the progression of the disease.

### Construction of state-transition AML state-space

To apply our state-transition model, we first create a state-space from the normal and AML RNA-seq data. Transcriptome states are identified in a state-space which is created for each dataset. The singular value decomposition (SVD) is applied to mean-centered gene expression data consisting of normal and primary, or newly diagnosed, AML peripheral blood samples as $\hat{X}=X-\bar{X}$ where $\bar{X}$ represents the mean gene expression. The SVD for $\hat{X}$ is given by

4 $$\hat{X}=U\Sigma {V}^{* }$$

where $U$ is a unitary matrix, $\Sigma$ is a diagonal matrix that contains the singular values, and the columns of the matrix ${V}^{* }$ correspond to the coefficient weights of each gene, referred to as “*eigengenes*” of each gene in the transcriptome^37^. The transcriptome state-space is modeled with the principal components and singular values of the data as ${PC}=U\Sigma .$ The principal component that resulted in the greatest separation of the normal and newly diagnosed AML peripheral blood samples was used to define the state-space^9,10^. Additional samples from each database, such as bone marrow or blood from recurrent disease samples were projected into the state-space using the eigengenes as follows. Given data matrix ${X}_{m}$, samples are mean-centered relative to the primary AML state-space as $\hat{{X}_{m}}={X}_{m}-\bar{X}$ and the projection of the new samples into the state-space is given by multiplying the data by the eigengenes, $P{C}_{m}=\hat{{X}_{m}}V.$ A state-space could not be constructed for the TCGA dataset because no normal blood or bone marrow samples were available at the time of this study, therefore, only surprisal analysis was performed on this subset of data.

### Feature selection for AML state-space

We used a feature selection method based on mutual information^38,39^ to increase the separation between normal and AML samples in the state-space^9^ (Box 1). Mutual information was calculated between the transcriptomes and a state indicator vector that consisted of binary values corresponding to normal or AML samples. After calculating all values of mutual information, gene transcripts were sorted based on mutual information scores. The distance between control and leukemia clusters was iteratively measured by removing genes that had the lowest mutual information. The separation was measured by the distance between the maximum value of the non-leukemia cluster and the minimum value of the leukemia cluster in the state-space. The SVD was then used on the selected features to create the state-space. There were 45,308 total unique sequenced transcripts for TARGET and 54,480 for BEATAML datasets. The number of selected genes for the BEATAML state-space was 1034 (Supplementary Table 10). The separation between normal and AML samples in the TARGET dataset was not increased with gene selection, therefore all sequenced genes were used for the TARGET AML state-space. No state-space was created for the TCGA dataset due to missing normal samples.

Box 1 Algorithm for creating a transcriptome state-space with mutual information **Construction of AML transcriptome state-space**

**Input**: transcriptome ${X}_{i}$ $(i=1,\ldots n)$ normal-AML samples $Y=\{0,1\}$

**Output**: a subset of transcriptome ${X}_{j}$, a set mutual information

$${\hat{I}}_{{all}}=\{\hat{I}\left({X}_{1}{;Y}\right),\ldots ,\hat{I}\left({X}_{i}{;Y}\right),\ldots ,\hat{I}\left({X}_{n}{;Y}\right)\}$$

**for** $i=1,\ldots ,n$ in ${X}_{i}$ **do**

${\hat{I}}_{{all}}[i]$ ← $\hat{I}\left({X}_{i}{;Y}\right),$ mutual information calculated based on algorithm of mixed random variable estimator^39^ given by $\hat{I}({X;Y})\equiv \frac{1}{n}{\sum }_{i=1}^{n}\log {\left(\frac{d{P}_{{XY}}}{d{P}_{X}{P}_{Y}}\right)}_{\left({x}_{i},{y}_{i}\right)}$

**end for**

sort ${\hat{I}}_{{all}}[i]$ in descending order, then store index to ${Xidx}[1,\ldots n$] from sorted ${\hat{I}}_{{all}}$

PC ← SVD for ${Xidx}[1,\ldots n]$

Normal cluster ← PC for normal samples

AML cluster ← PC for leukemia samples

**while** sup{sgn(sup{AML cluster} − sup{normal cluster}) ∗ normal cluster} > inf{sgn(sup{AML cluster} − sup{normal cluster}) ∗ AMLcluster}

**do**

PCs ← SVD for ${Xidx}[1,\ldots n]$

Normal cluster ← PC for normal samples

AML cluster ← PC for leukemia samples

$$n$$ ← $$n-1$$

end while

### Calculation of state-transition critical points

To calculate critical points for the state-transition model, we first identified the critical points ${c}_{1}$ and ${c}_{3}$ associated with normal and AML states using the centroids of two clusters from k-means clustering (k = 2) in the state-space. To estimate the unstable state, ${c}_{2}$, we constructed several leukemia potentials, ${U}_{p}$, with values of ${c}_{2}$ ranging from ${c}_{1}$ to ${c}_{3}$. Then, the Boltzmann ratio with fixed temperature and Boltzmann constant was calculated for each potential

5 $$\Pr ({c}_{3})/\Pr ({c}_{1})=\exp \left({U}_{p}\left({c}_{1}\right)-{U}_{p}\left({c}_{3}\right)\right)$$

The value for ${c}_{2}$ was chosen such that the observed $\left(\frac{\Pr \left({c}_{3}\right)}{\Pr \left({c}_{1}\right)}\right)$ and theoretical ratios of the number of samples in ${c}_{1}$ and ${c}_{3}$ agreed, so that the observed and theoretical ratios were equal,

6 $$\Pr ({c}_{3})/\Pr ({c}_{1})=\exp \left({U}_{p}\left({c}_{1}\right)-{U}_{p}\left({c}_{3}\right)\right).$$

All samples in the state-space were associated with a critical point based on the minimum distance from the sample to the nearest critical point ${c}_{1}$, ${c}_{2}$, or ${c}_{3}$.

### Pseudotime for state-transition model

**Fig. 6:**
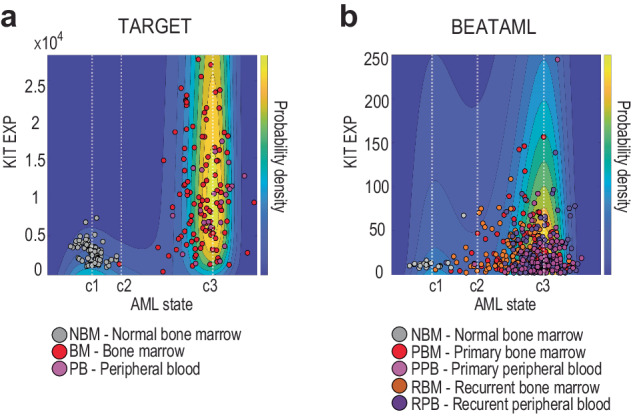
AML state-space with KIT expression as pseudotime. **a**, **b** Probability density predicted by the Fokker–Planck equation in the AML state-space with state-transition critical points using KIT expression level as a pseudotime marker.

Because the samples were taken from single timepoints from different individuals, we used a pseudotime approach to infer disease dynamics based on the ensemble of data. We identified KIT (ENSG00000157404) and CD33 (ENSG00000105383) which are known to be involved in leukemogenesis^40,41^ as candidate genes to define pseudotime. These genes were selected based on their reported association with AML and because they were present in all samples. We observed relatively high expression of CD33 in normal samples, providing poor differentiation between normal and AML groups. We found via t-test analysis that KIT expression between normal and AML samples was significantly different for both TARGET (p < 0.01) and BEATAML (p < 0.01). CD33 expression between normal and AML samples was significant in BEATAML (p = 0.012), but not TARGET (p = 0.30). We therefore used KIT as a pseudotime gene marker (Fig. 6).

### Surprisal analysis

Surprisal analysis^5,6^ was used to identify the transcriptome steady state of each sample as well as deviations from the steady state. Steady state is a reference biological state linked with the most stable distribution of mRNA molecules, or transcripts. SA determines the steady state by calculating the theoretically expected distribution of mRNA species for each AML sample. The approach assumes that any tissue, healthy or diseased, reaches a state of minimal free energy at a given temperature and pressure, subject to environmental and genomic constraints. A constraint is a physical or molecular process that increases the free energy of the system. Constraints are identified by examining how the observed levels of each gene transcript deviate from their levels at the steady state at each time point or sample. Transcripts deviating from the steady state in a coordinated manner are grouped to identify unbalanced processes^6,7^.

Using the following equation, SA uncovers the steady state and all the constraints in the sample *k*:

7 $${\ln({X}_{i}(k))=\ln({X}_{{io}}(k))-\mathop{\sum}\limits_{\alpha =1}{G}_{i\alpha }{\lambda }_{\alpha }\left(k\right)}$$

where *i* is the transcript of interest, $l{nX}_{{io}}\left(k\right)$ is the natural logarithm of gene expression level in a sample *k* when the sample is at the steady state free of constraints and ${\sum }_{\alpha =1}{G}_{i\alpha }{\lambda }_{\alpha }\left(k\right)$ represents the sum of deviations in the expression level of the gene *i* due to the various constraints. More details on theory is provided in references^5,6^.

The term ${G}_{i\alpha }$ denotes the degree by which transcript *i* is influenced by unbalanced process *α*. Transcripts are grouped into biological processes based on ${G}_{i\alpha }$ values^7^. The sign of each ${G}_{i\alpha }$ indicates the correlation or anti-correlation between co-expressed transcripts in the same process. The term ${\lambda }_{\alpha }\left(k\right)$ represents an amplitude, or relative importance, of an unbalanced process *α* in sample *k*. All calculated ${\lambda }_{\alpha }\left(k\right)$ and ${G}_{i\alpha }$ values are provided in Supplementary Tables 1, 2. The algorithm for calculating the amplitudes of the unbalanced processes is presented in Box 2. A detailed step-by-step mathematical procedure of SA can be found in the supplementary file of Vasudevan et al.^6^. Transcripts with significant weights ${G}_{i\alpha }$ (Supplementary Tables 3, 4 and 5) are grouped using Gene Ontology^42,43^ to provide a biological interpretation of each process.

To examine the number of significant processes in the dataset we check how many processes are required to reproduce the experimental data as previously described^6^. Threshold limits for ${\lambda }_{\alpha }\left(k\right)$ were calculated using standard deviations of the levels of 1% of the most stable transcripts in the datasets. Only processes which were above the threshold limits were included in a patient-specific barcode and included in the calculation of a deviation from the steady state. The term ${\sum }_{\alpha =1}{G}_{i\alpha }{\lambda }_{\alpha }\left(k\right)$ is the deviation from the steady state per transcript molecule. To calculate free energy change per sample, *k*, relative to the steady state, we compute

8 $$\frac{{FEC}}{{RT}}\equiv \sum _{i=1} X_{i} \sum_{\alpha =1}{G}_{i\alpha }{\lambda }_{\alpha }(k)$$

for each *k*. Free energy changes were then related to critical points (${c}_{1}$, ${c}_{2}$, ${c}_{3}$) from the state-transition model.

Box 2 Algorithm for identifying the amplitude of the unbalanced processes $({\lambda }_{\alpha }\left(k\right))$ and the weight of the transcripts $(G_{i\alpha })$ using surprisal analysis

**Identification of unbalanced process amplitudes (**${{\boldsymbol{\lambda }}}_{{\boldsymbol{\alpha }}}\left({\boldsymbol{k}}\right)$ **) and gene weights** ${{\boldsymbol{(G}}}_{{\boldsymbol{i}}{\boldsymbol{\alpha }}}{\boldsymbol{)}}$

**Input: Transcriptome Data (I** = **1, …, n),**

[G,W,V] = svd(log(Data));

rows = size(Data,1);

columns = size(Data,2);

if rows>columns

L = V*W(1:columns,:);

end

if rows<columns

W0 = zeros(columns-rows,columns);

WW = [W; W0];

L = V*WW;

end

**Output: G** = $${G}_{i\alpha }$$ **, L** = $${\lambda }_{\alpha }\left(k\right)$$

### Barcode calculation

Barcodes are schematic representations of the patient-specific signaling signatures (PaSSS)^28^. Unbalanced processes with amplitudes which exceeded threshold limits were included in the barcodes. Threshold limits for ${\lambda }_{\alpha }\left(k\right)$ values^6,28^ ${\lambda }_{\alpha }\left(k\right)$ (*α* = 1, 2, 3… n) were discretized into barcodes as follows: for each $\alpha ,$ if ${\lambda }_{\alpha }\left(k\right)$ > error limit then it is discretized to 1; if ${\lambda }_{\alpha }\left(k\right)$ < − error limit then it is discretized to −1; and if −error limit < ${\lambda }_{\alpha }\left(k\right)$ < error limit then it is discretized to 0.

### Generation of unbalanced process subnetworks

The STRING database^44^ was used to define functional connections between transcripts which were found to be influenced by the unbalanced processes. Visualizations of subnetworks based on STRING parameters were generated using Cytoscape^45^ software. Signs of ${G}_{i\alpha }$ were used to distinguish between correlated and anti-correlated transcripts. The ${G}_{i\alpha }$ values corresponded to the circle radiuses representing the transcripts in the process. The product of the gene weight and the process amplitude, ${G}_{i\alpha }{\lambda }_{\alpha }\left(k\right)$ indicates the amount of deviation in expression level of a transcript *i* from its reference state due to process *α*. Positive values of ${G}_{i\alpha }{\lambda }_{\alpha }\left(k\right)$ indicate an increase relative to the steady state, and negative values indicate a reduction (Fig. 4, Supplementary Figs. 6 and 7).

### Connection between surprisal analysis and state-transition modeling via the singular value decomposition

Surprisal analysis and the state-transition model share common mathematical features via utilization of the SVD. In SA we quantify first the co-variance matrix of natural logarithms of protein expression levels as dictated by the theory^5^ and then fit it into Eq. 7 to quantify the expected transcript levels at the steady state and deviations thereof in all examined samples. The logarithm of the measured expression is used to relate the RNA concentration to the chemical potential using fundamental physical–chemical relationships^30^. In practical terms, a matrix containing the natural logarithm of transcripts^6^ is used as an intermediate step, which calls for the construction of two square, symmetric, co-variance matrices. One is smaller with a maximal rank equivalent to the number of samples and the second is larger, equivalent to the number of transcripts. These matrices are diagonalized to calculate eigenvectors and eigenvalues using the SVD. Eigenvectors and eigenvalues are used to calculate the amplitudes of the processes: ${\lambda }_{\alpha }\left(k\right)$ for each sample and ${G}_{i\alpha }$ values (Box 2 and supplementary information of Vasudevan et al.^6^). Similarly, the state-space for the state-transition model is created via the SVD from the log transformed data matrix, however, the data is first mean-centered ($\hat{X}$). The state-space is then constructed from one or more left singular vectors from the SVD which maximizes the separation between the normal and AML samples and is used to estimate state-transition critical points.

### Reporting summary

Further information on research design is available in the Nature Research Reporting Summary linked to this article.

### Supplementary information

Supplementary file

Reporting summary

Supplementary Table 1

Supplementary Table 2

Supplementary Table 3

Supplementary Table 4

Supplementary Table 5

Supplementary Table 6

Supplementary Table 7

Supplementary Table 8

Supplementary Table 9

Supplementary Table 10

## Data Availability

The study’s data are all publicly available and are included in the article or Supplementary Materials.

## Code availability

All equations and codes used in this article are detailed in the Methods section and/or referenced.
