# Supplementary material for: Transcriptome free energy can serve as a dynamic patient-specific biomarker in acute myeloid leukemia
Source: NPJ Syst Biol Appl. 2024 Mar 25;10:32. doi: 10.1038/s41540-024-00352-6 (PMC10963775; doi:10.1038/s41540-024-00352-6)
Supplement: Supplementary file 1 — Supplementary file [file 41540_2024_352_MOESM1_ESM.pdf]

Transcriptome free energy can serve as a dynamic patient-specific biomarker in acute myeloid leukemia

Lisa Uechi[1#], Swetha Vasudevan[2#], Daniela Vilenski[2], Sergio Branciamore[1], David Frankhouser[1], Denis O'Meally[2,3], Soheil Meshinchi[4], Guido Marcucci[5], Ya-Huei Kuo[5], Russell Rockne[1]*, Nataly Kravchenko-Balasha[2]*

Supplementary material

1. Supplementary Note 1. Biological interpretation of unbalanced processes in AML datasets
2. Supplementary Note 2. Biomarkers in AML
3. Supplementary Figures (1- 12)
4. Supplementary References

Supplementary Note 1. **Biological interpretation of unbalanced processes in AML dataset**

Interleukin-related unbalanced processes Different types of interleukins (ILs) have been shown to play an important role in the survival, growth and apoptosis of leukemia cells[1,2]. We found that many IL related genes were involved in different unbalanced processes. For example, IL1 related signaling pathways, which acts as an autocrine growth factor in AML cells[3], was found in different processes of the three datasets (**Supplementary tables 3-5**): At least ~9% of the patients harbored at least one of the processes which included IL1 signaling pathway, namely processes 3+ , 9- or 12- in the TARGET dataset.

It is important to note that the sign of the process (e.g., +, positive) indicates the correlation or anti-correlation between the same processes in different tumors. For example, if the process α is assigned the values: $\lambda\alpha(1) = 29.1$, $\lambda\alpha(20) = 0.0$, and $\lambda\alpha(138) = 22.5$, it means that this process influences the tumors of the samples indexed 1 and 138 in the same direction, while it is inactive in patient 20. This also means that transcripts with positive $G_{i\alpha}$ values will be upregulated in patient 1 and 138 since the partial deviation in expression level of the transcript *i* due to unbalanced process α *is* calculated using $G_{i\alpha}\lambda_{\alpha}(k)$. Therefore transcripts with negative $G_{i\alpha}$ values will be downregulated in this particular sample due to process α.

Datasheets in **Supplementary Tables 3-5** are named G_{α} *(positive) or* G_{α} *(negative)* to divide anticorrelated transcripts (transcripts with negative $G_{i\alpha}$ values are anticorrelated to the transcripts with negative $G_{i\alpha}$ values in each process α) in each process. To calculate whether a particular transcript or gene category upregulated or downregulated in a sample α, $G_{i\alpha}\lambda_{\alpha}(k)$ is calculated as explained above. Thus, is we state "At least ~9% of the patients harbored at least one of the processes which included IL1 signaling pathway, namely processes 3+, 9- or 12- in the TARGET dataset." Meaning that 9% of AML samples have upregulated IL1 signaling pathway that can be found in processes 3 (within the list of transcripts with significant positive $G_{i\alpha}$ values, and $\lambda_3(k)$ values are positive in these samples), in process 9 (within the list of transcripts with significant negative $G_{i\alpha}$ values, and $\lambda_9(k)$ values are negative in these samples) and in process 12 (within the list of transcripts with significant negative $G_{i\alpha}$ values, and $\lambda_{12}(k)$ values are negative in these samples).

Using the explanation above we continue the biological description of unbalanced processes found in AML samples:

~22% of the patients in TCGA dataset harbored at least one of the processes 2+, 4+, 6- and 12+, which included IL1 pathway as well. Similarly, ~20% of the patients in BEATAML dataset harbored one of the IL1+ processes, namely 2+,4+, 7+ or 9-. Processes 9- and 12- in the TARGET dataset and process 9- in the BEATAML dataset was found to be active only in the cancer patients (c_3).

IL2 stimulates T-lymphocytes to produce IL3 thereby contributing to proliferation of AML cells[1]. Genes related to IL2 signaling pathway was found in at least four processes in each dataset: namely in processes 3+, 5-, 9- and 11- in TARGET, processes 2+, 3+, 9-, 11-, 12- and 13+ in TCGA and processes 2-, 5+, 6- and 12+ in BEATAML. Processes 5-, 9- and 11- in TARGET and process 12+ in BEATAML were significant only in cancer patients (c_2 and c_3).

IL8 plays an important role in the relapse or refractory AML. It is often associated with poor prognosis and helps to promote AML cell growth and chemo resistance[4]. Genes involved in IL8 signaling pathway were found in 9 out of the 12 unbalanced processes (all except processes 2, 6 and 11) in TARGET dataset, out of which processes 1+, 3+ and 4+ were found in both cancer and normal patients. The other 6 processes – 5+, 7-, 8+, 8-, 9+, and 12- were found only in cancer patients (c_2 and c_3). More than 35 % of patients in TARGET dataset had processes with induced genes related to IL8 pathway.

Another IL found to be involved in AML progression is IL10. IL10 along with E-Cadherin, an adhesion molecule, help to promote AML cell survival[5]. Genes related to IL10 pathway were found in processes 2+, 3+, 9- and 11 -in TARGET, processes 2+, 4-, 6+, 9-, 10+ and 14- in TCGA and processes 2+, 3+, 5+ and 8- in BEATAML datasets. Processes 9- and 11- in TARGET and process 8- in BEATAML were found only in cancer patients (c_2 and c_3). More than 13% of patients, in both TARGET and TCGA dataset, and 20% of patients in BEATAML dataset were found to have these processes.

Other dominant biological categories in unbalanced processes Apart from the interleukin related processes, another biological processes specific to AML, were also found (**Supplementary Tables 3-5).** The most common biological process was related to leukocyte migration. Genes involved in this biological pathway were found in processes 4+, 5+, 9- and 12- in TARGET, 15 out of 18 processes of TGCA (all except processes 6, 7 and 17) and 11 out of 14 processes of BEATAML (all except processes 10, 11 and 14). Here processes 5+, 9- and 12- in TARGET and processes 8-, 9+ and 12+ in BEATAML were found *only* in cancer patients (c_2 and c_3). Leukocyte migration along with IL1 and tumor necrosis factor (TNF) (another biological process which was induced by co expressed genes in different unbalanced processes) play an important role in developing leukostasis[6,7]. Leukostasis is a condition in AML patients where the WBC plugs are seen in the microvasculature. It's associated with adverse prognosis and can be life threatening without prompt treatment. Genes expressing TNF were also found in processes 4+, 5-, 6-, 8+ and 10+ in TARGET, 1+, 6-, 8+, 9-, 10+, 12-, 15+ and 17+ in TCGA and 2+, 4+ and 9+ in BEATAML. Processes 5-, 8+ and 10+ in TARGET and process 9+ in BEATAML were found only in cancer samples (c_2 and c_3).

Additional signaling pathways were also found in the unbalanced processes. PI3K signaling, ERK 1 and ERK 2 signaling pathways, MAPK cascade, NF-kappa signaling, were among the signaling pathways which were commonly found in the unbalanced processes of different datasets. PI3K signaling is associated with survival during cellular stress and therapeutic resistance[8,9]. It was induced in processes 1+, 2+, 9- and 9+ in TARGET, processes 1+, 3+, 4+, 4-, 5-, 9-, 10-, 14- and 17+ in TCGA and processes 3+, 5+, 6+, 9+, 9- and 12+ in BEATAML. Process 9+/- in TARGET and processes 9+/- and 12+ in BEATAML were found only in cancer samples (c_2 and c_3).

MAPK cascade includes both ERK 1 and 2 and P38 MAPK pathways. They are associated with expansion and survival of leukemic clones[10,11]. Processes 1+, 1-, 2+, 3+, 3-, +, 7-, 8+ and 12- in TARGET, processes 1+, 2+, 3+, 4+, 4-, 5-, 8+, 8-, 10+, 11+, 11-, 12+, 12-, 13+, 14-, 17+ in TCGA and processes 3+, 4+, 5+, 6+, 6-, 7+, 7-, 8-, 9-, 11- and 14- in BEATAML had genes involved in MAPK cascade. Processes 7-, 8+ and 12- in TARGET and processes 8-, 9- and 14-

in BEATAML were found only in cancer samples (c_2 and c_3). Genes playing a role in NF-kB signaling pathway were found in processes 2+, 3+, 4+, 5+ and 7- in TARGET, processes 1+, 2+, 6-, 8+, 12-, 13+, 14- and 17+ in TCGA and processes 1-, 4+, 5+, 7+, 8+- and 11- in BEATAML. Processes 5+ and 7- in TARGET and process 8+ in BEATAML were found only in cancer samples (c_3). Activation of NF-kB allows leukemic cells to avoid programmed cell death and increase cell proliferation[12].

Supplementary Note 2. Biomarkers in AML

S100A9 is one of the most over expressed genes in certain subtypes of AML[13]. It is a calcium binding protein, which stimulates the recruitment of myeloid cells and myeloid-derived suppressor cells, leading to tumor growth and metastasis[14–16]. S100A9 is a potential differentiation agent in myelomonocytic and monocytic AMLs[17].

CD34 is an important prognostic biomarker in AML[18]. This stem cell marker is associated with methylation and is found at high levels, especially in pediatric patients, in many cancer types, including AML[19].

FUT4 is a fucosyltransferase, which catalyzes fucosylation (adding a fucose residue to N-glycans, O-glycans and glycolipids), a post-translational modification widely existing in proliferating cancer cells[20,21]. High levels of FUT4 can be associated with good prognosis in AML[22].

HOMER3 belongs to a family of cytoplasmic scaffolding proteins, which can regulate transcription and play an important role in the differentiation and development for some tissues[23–26]. Its overexpression is associated favorable cytogenetics in AML[27].

MEIS1 was first identified in a murine leukemia model. It functions in regulating growth and differentiation during vertebrate development[28,29]. High MEIS1 expression levels are associated with resistance to conventional chemotherapy[30].

JAG1, one of the five canonical ligands for Notch receptors, belongs to the Serrate/Jagged family [31,32].JAG1 plays an important role in both physiological and pathological conditions, including embryonic development and cancer. High JAG1 expression levels is an independent favorable prognostic factor in patients with AML[33].

CD99 is a cell surface glycoprotein known to be involved in migration of neutrophils, T-cell adhesion, and T-cell death via caspase-independent pathway. In cancer cells high CD99 expression levels were found to be correlated with tumor invasiveness and with lower survival rates. Studies show that this gene plays an important role in cell survival and cell migration in AML[34].

Cyclin-dependent kinase 6 (CDK6) is a regulatory enzyme of the cell cycle that plays an important role in leukemogenesis and the maintenance of leukemia stem cells (LSC). High expression levels of CDK6 show an adverse effect on OS (overall survival) of certain subset of AML patients[35].

STMN1 is a cytosolic protein that mediates microtubule destabilization[36]. This protein acts as a marker of PI3K pathway activity and plays an important role in cell proliferation, differentiation, and survival in AML[37–39].

FLT3 is a membrane-bound receptor which is expressed in hematopoietic progenitor cells[40]. It regulates differentiation and proliferation of hematopoietic cells. Overexpression of FLT3 gene with or without mutation was found to be associated with poor prognosis in AML patients[41,42].

CEBPA belongs to the transcription factors family and plays an important role regulating the genes involved in cell cycle and body weight homeostasis[43–46]. Studies suggest that AML patients with high CEBPA expression levels have a favorable prognosis[47,48].

BIK is a proapoptotic, BH3-only gene within the BCL2 family and is found at high levels in AML patients[49]. Its high expression levels are associated with abnormal cytogenetics[50].

CAT encodes catalase, a key antioxidant enzyme in the body's defense against oxidative stress. Studies show that high CAT expression is correlated with positive response to standard AML therapy using cytosine arabinoside plus daunorubicin[51].

PBX3 belonging to the HOX family, plays an important role in initiation of AML[52]. It also promotes cell proliferation and is associated with resistance to chemotherapeutic agents and adverse prognosis in patients[53].

Supplementary Figures

Supplementary figure 1

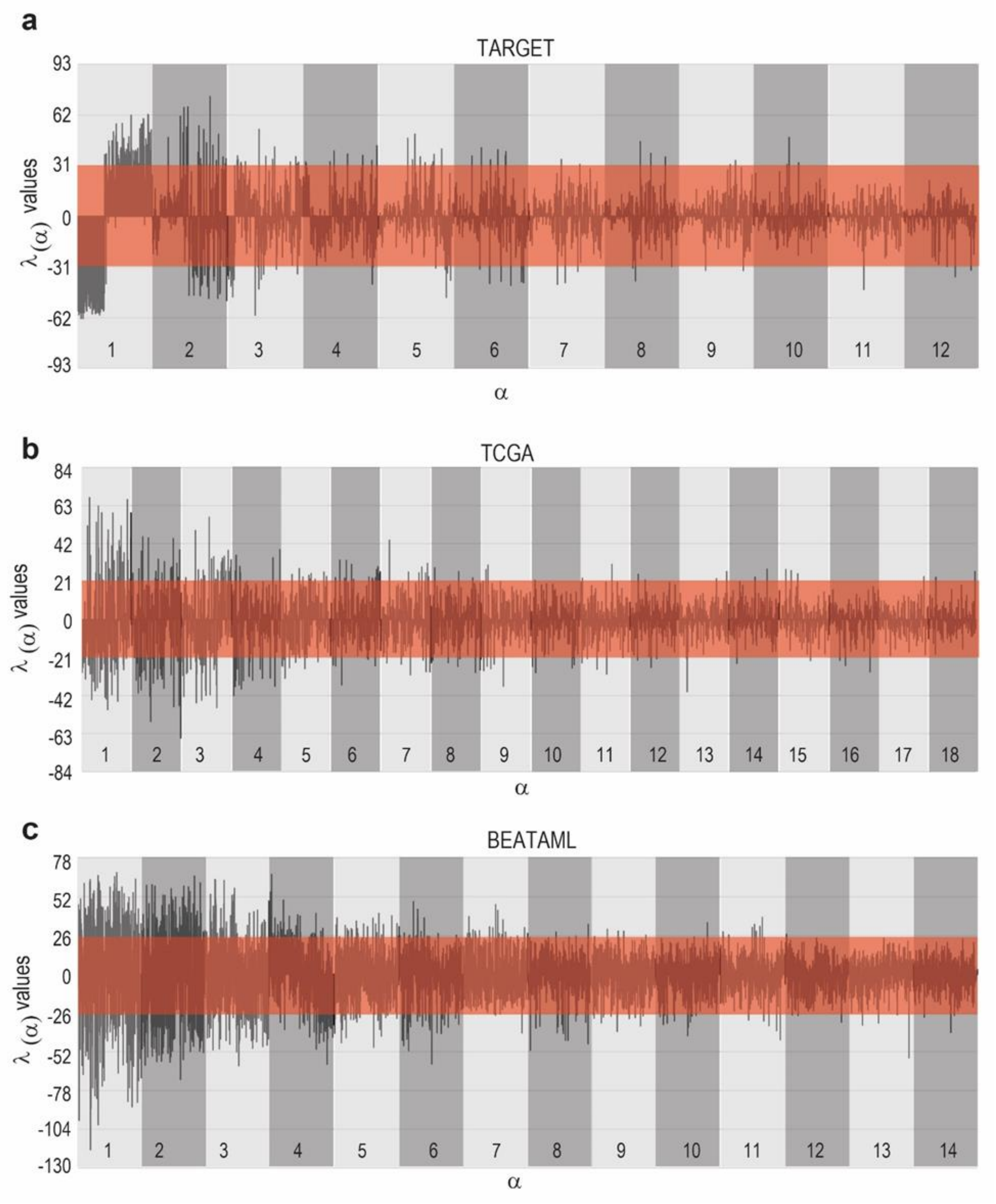

Supplementary figure 1. Amplitudes representing the significance of each unbalanced process α in each sample found in Target, BeatAML and TCGA datasets are presented. Red horizontal boxes indicate error limits. (**a**) Amplitudes ($\lambda(\alpha)$) values are provided for each of 210 samples for 12 unbalanced processes found in the Target dataset. Each grey box (1-12) includes 210 λα values. (**b**) Amplitudes (λα) values are provided for each of 151 samples for 18 unbalanced processes found in the TCGA dataset. Each grey box (1-18) includes 151 ($\lambda(\alpha)$) values. (**c**) Amplitudes (λα) values are provided for each of 497 samples for 14 unbalanced processes found in the BeatAML dataset. Each grey box (1-14) includes 497 ($\lambda(\alpha)$) values. In all datasets, amplitudes decrease as index α increases.

Supplementary figure 2

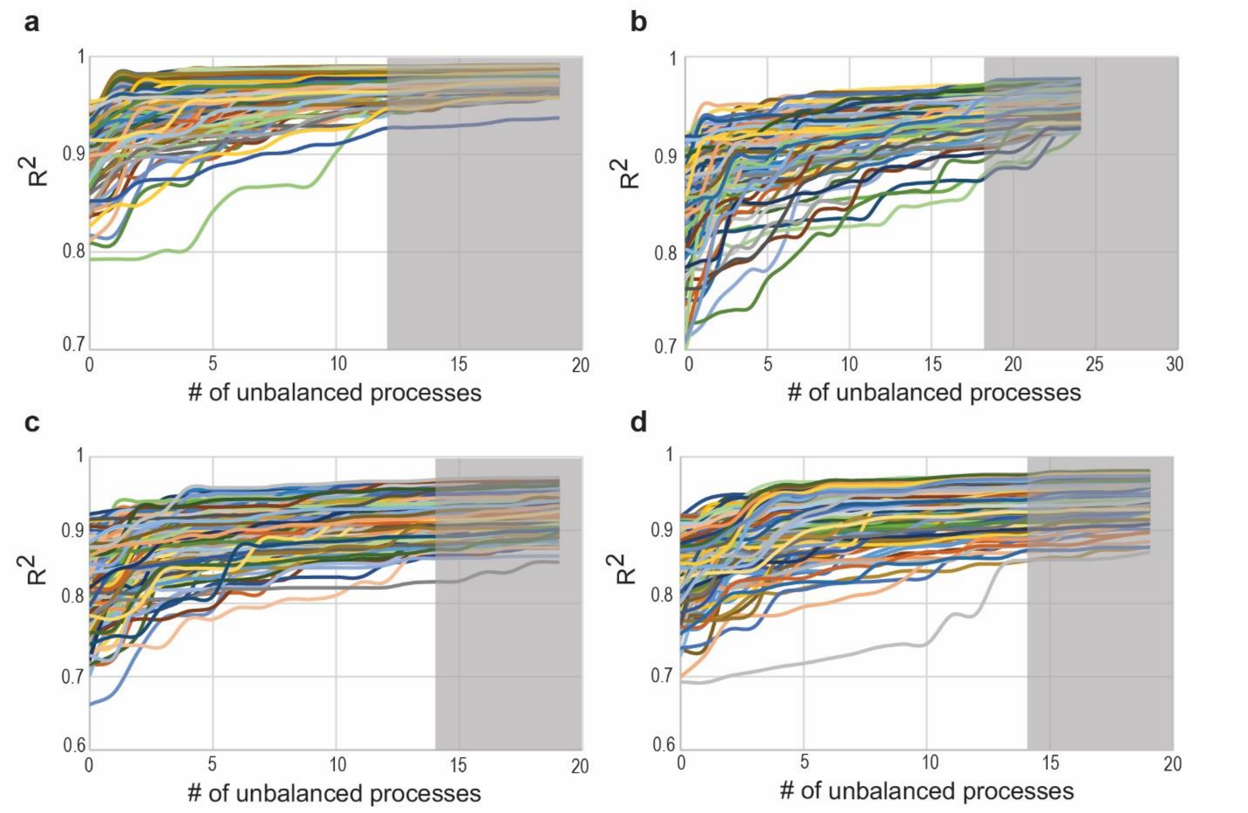

Supplementary figure 2 To identify the number of unbalanced processes we first calculate how many processes are required to reproduce the experimental data. R^2 values show the correlation between the experimental data and the theoretical calculations and calculated for all the patients in all 3 datasets. (**a**) The R^2 values for all the 190 patients in TARGET dataset are shown. The R^2 values reach a plateau after process 12, showing 12 processes are enough to characterize the dataset. (**b**) The R^2 values for all the 151 patients in TCGA dataset are shown. The R^2 values reach a plateau after process 18, showing 18 processes are enough to characterize the dataset. (**c,d**) The R^2 values for 225 patients (**c**) and for the rest 263 patients (**d**) in BEATAML dataset are shown. The R^2 values reach a plateau after process 14, showing 14 processes are enough to characterize the dataset. In addition, we calculate threshold limits for $\lambda_\alpha(k)$ values as detailed in [54,55]. These two methods are utilized to define the final number of processes in each dataset.

Supplementary figure 3

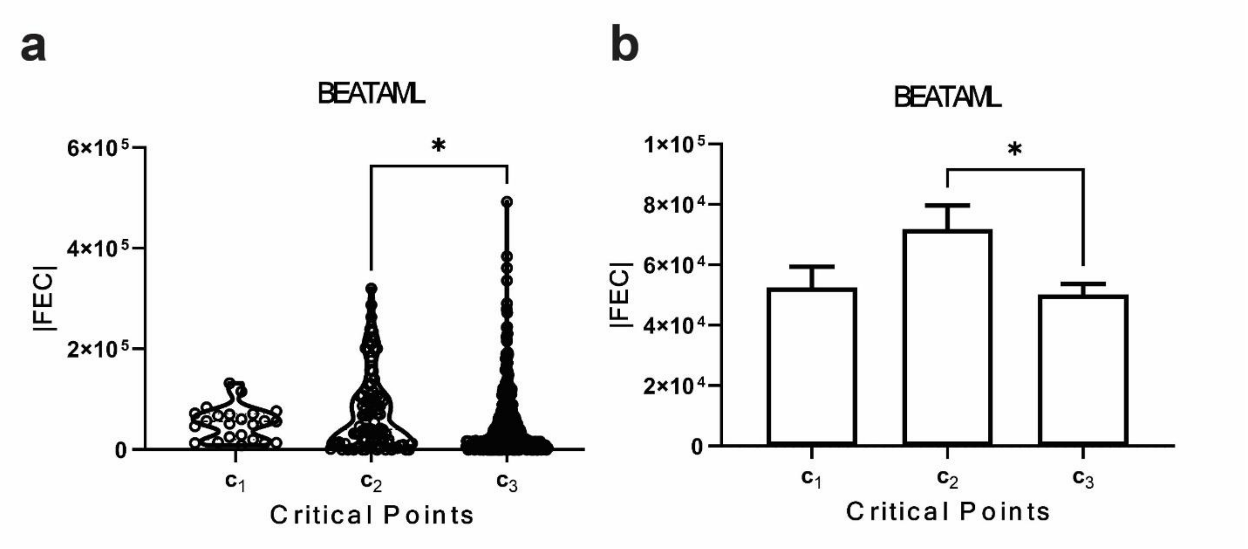

Supplementary figure 3. Transcriptome free energy changes are associated with state-transition critical points for BEATAML dataset. (a) We observed higher mean FEC at the unstable transition point (c_2) with significant differences between c_2 and c_3 (p=0.016). Despite a clear tendency towards higher FEC values in c_2 the difference in FEC between c_1 and c_2 was not statistically significant due to the inclusion of normal samples with high expression of NPM1. (**b**) The box plot shows the mean values and std errors of FEC of BEATAML by critical points. *Two samples were excluded from c_1 as detailed in Supplementary table 8.

Supplementary figure 4

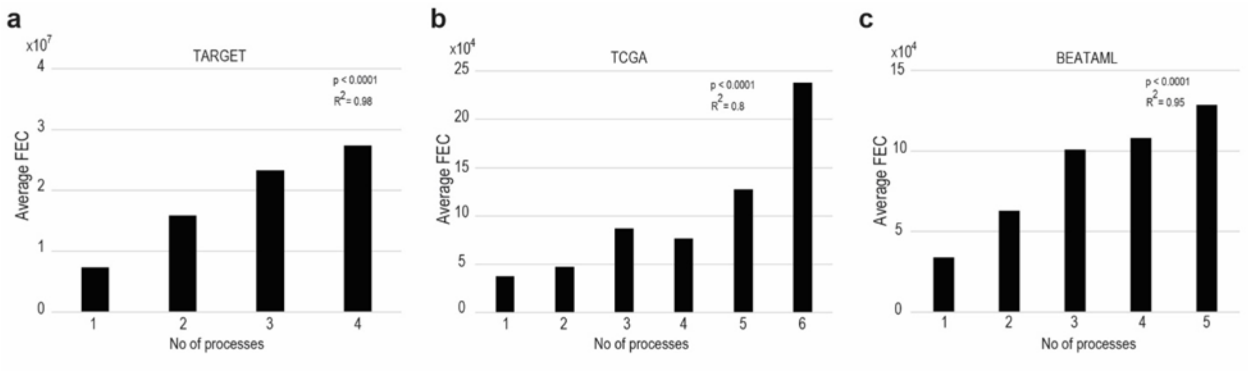

Supplementary figure 4. Strong correlation is observed between the FEC values and the number of processes in all datasets. The average FEC values of all patients having 1, 2, 3…, n processes were plotted for (**a**) TARGET (**b**) TCGA (**c**) BEATAML datasets. The R^2 values show an excellent correlation between the FEC and the number of processes. The p values were calculated using Anova test and were found to be highly significant in all the datasets. Three samples (one sample in TCGA with 7 processes and two samples in TARGET with 6 processes) were not included in the calculations.

Supplementary figure 5

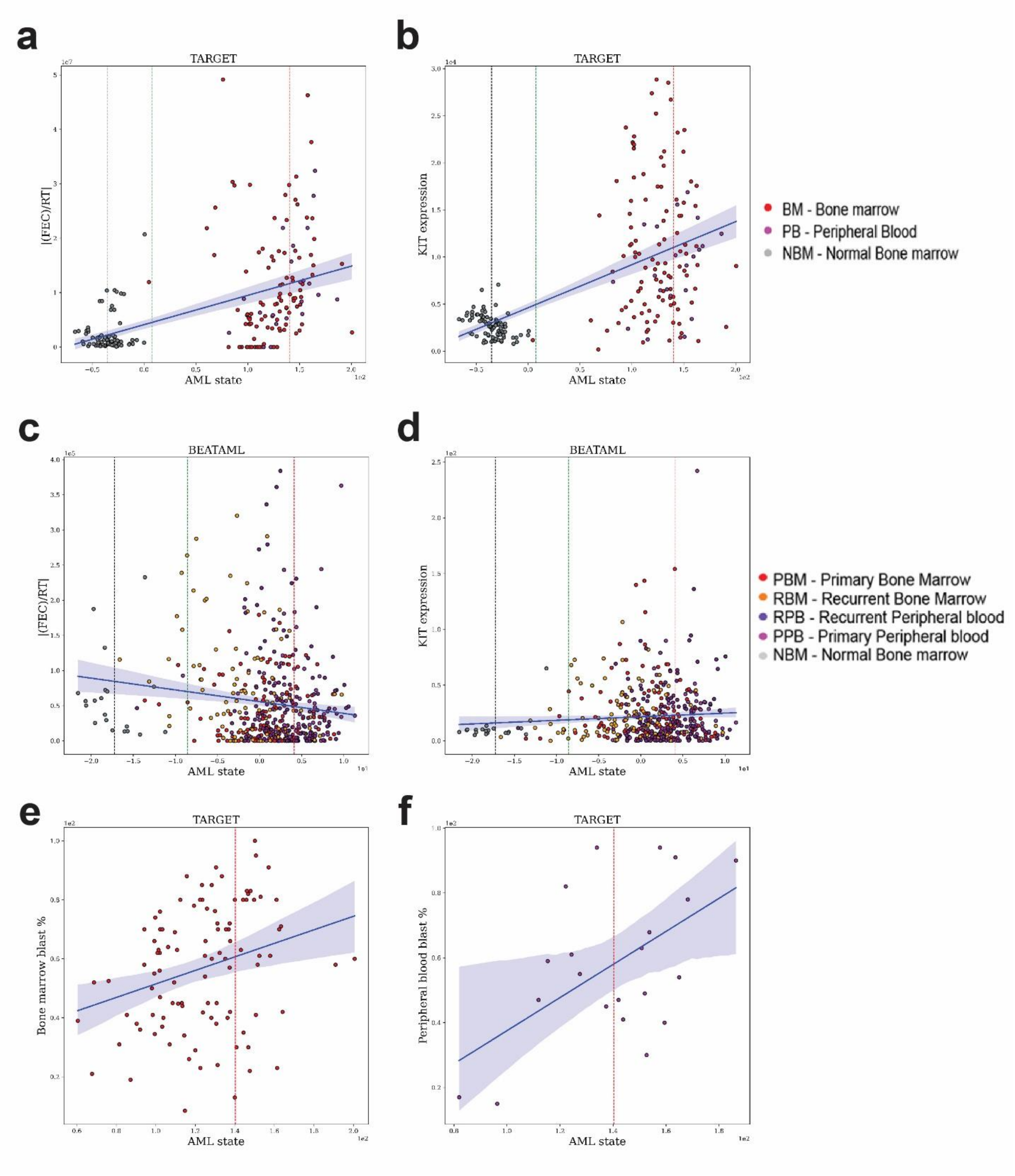

Supplementary figure 5. **Correlation analysis among AML states, FEC, KIT expression level, and blast percentages for TARGET and BEATAML.** Pairwise correlations between AML state and FEC for TARGET (**a**) and BEATAML (**c**), AML state and KIT expression level for TARGET (**b**) and BEATAML (**d**) are shown. The R-score and p-value between KIT expression level and

AML state for BEATAML are R=0.07 and p>0.05. Blast percentages of bone marrow and peripheral blood are available for TARGET dataset, and the pairwise correlations between Bone marrow blast percentage and AML state (**e**), peripheral blood blast percentage and AML state (**f**) are shown. The black, green, and red dotted horizontal lines correspond to c_1, c_2, and c_3, respectively.

Supplementary figure 6

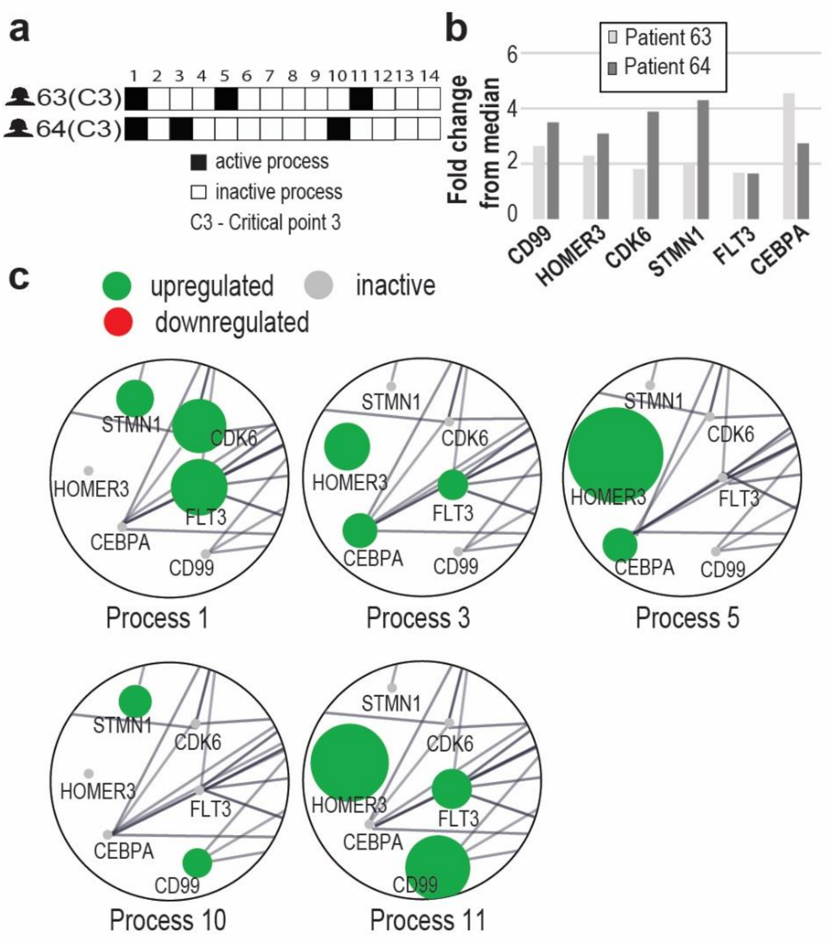

Supplementary figure 6. Similar gene expression levels in different patients may be attributed to different unbalanced processes in BEATAML dataset. (**a**) Barcodes of two AML patients (Primary Peripheral blood samples) are shown. These patients were classified to c_3 by ST analysis. Patient 63 was found to harbor processes 1, 5 and 11, while patient 64 has processes 1, 3 and 10. (**b**) The fold changes of six selected AML biomarkers are shown. CDK6, CD99, STMN1, HOMER3, FLT3 and CEBPA were up-regulated in both patients relative to their median expression levels across 488 patients. (**c**) Zoom images of unbalanced processes, comprising the barcodes of patients 63 and 64 (**a**) and in which the selected biomarkers whose levels are most influenced by those processes, are shown. Green denotes up-regulation due to a process α, red denotes down-regulation due to a process α, and gray denotes no change due to the process. Size of the biomarkers are relative to their weights in each process. Functional connections are according to STRING database.

Supplementary figure 7

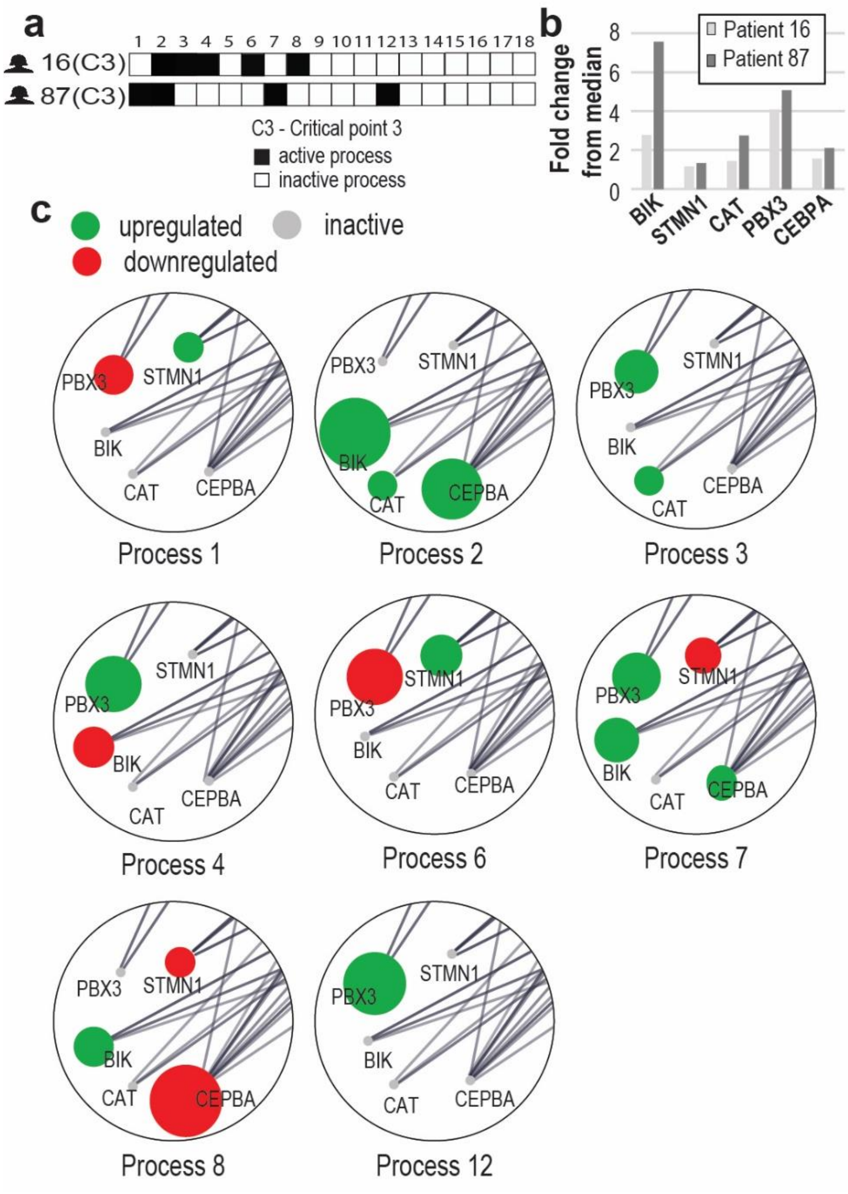

Supplementary figure 7. **Similar gene expression levels in different patients may be attributed to different unbalanced processes in TCGA dataset**. (**a**) Barcodes of two AML patients (Peripheral blood samples) are shown. These patients were classified to c_3 by ST analysis. (**b**) The fold changes of five selected AML biomarkers are shown. BIK, STMN1, CAT, PBX3 and CEBPA were up-regulated in both patients relative to their median expression levels across 151 patients. (**c**) Zoom images of unbalanced processes, comprising the barcodes of patients 16 and 87 (**a**) and in which the selected biomarkers whose levels are most influenced by those processes, are shown. Green denotes up-regulation due to a process *a*, red denotes down-regulation due to a process *a*, and gray denotes no change due to the process. Size of the biomarkers are relative to their weights in each process. Functional connections are according to STRING database.

Supplementary figure 8

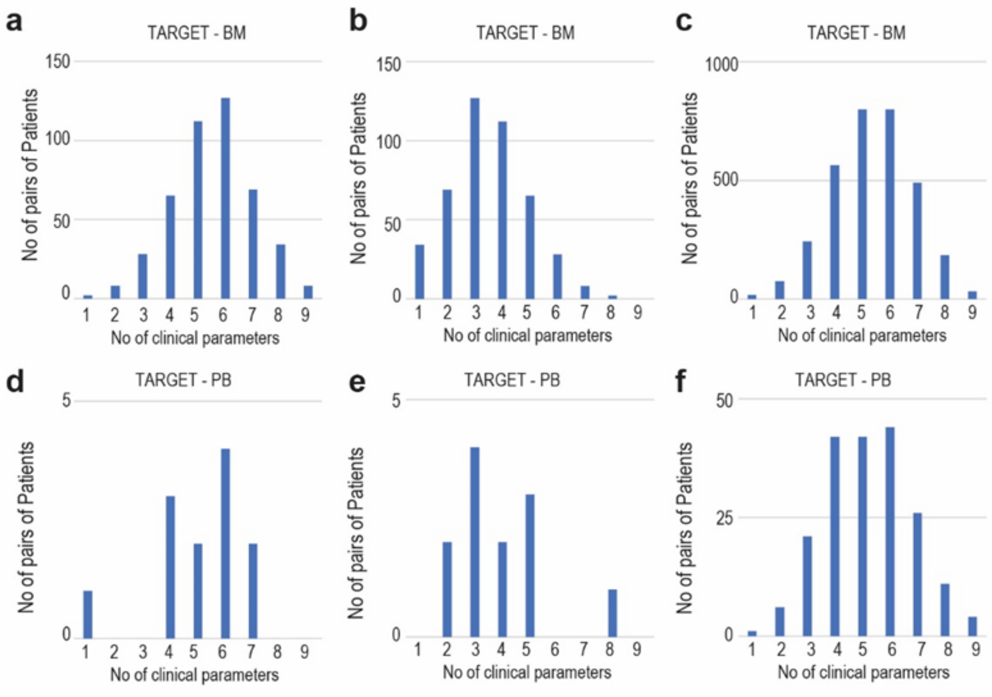

Supplementary figure 8. Comparison of number of pairs of patients with similar/different barcodes and number of similar clinical features. Plots showing the number of pairs of patients on the y axis and the number of similar clinical data on the x axis in TARGET dataset. Patients with similar PaSSSs and similar clinical data are shown in (**a**) and (**d**) for Bone marrow (BM) and Peripheral blood samples (PB). Patients with similar PaSSSs and different clinical data are shown in (**b**) and (**e**) for Bone marrow (BM) and Peripheral blood samples (PB). Patients with different PaSSSs and similar clinical data are shown in (**c**) and (**f**) for Bone marrow (BM) and Peripheral blood samples (PB). The patients were grouped based on the following criteria for each clinical data – age: 0-12 years/13-18 years/18+ years. Gender: male/female. FLT3 mutation: present/absent. Treatment: Chemotherapy/Targeted therapy/both. PB blasts: >2/ <2 (where 2 is the fold values from healthy values). BM blasts: >2/ <2 (where 2 is the fold values from healthy values). BM blast %: >2/ <2 (where 2 is the fold values from healthy values). WBC count: >2/ <2 (where 2 is the fold values from healthy values). CNV status: CBL deletion/ trisomy 8/ trisomy 21/ none.

Supplementary figure 9

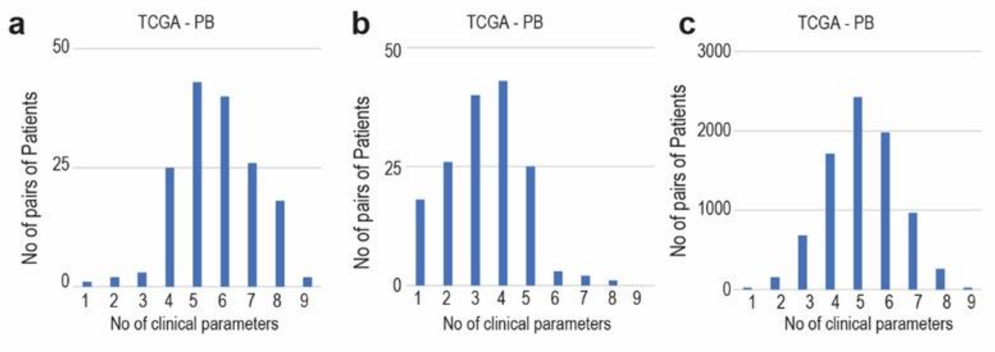

Supplementary figure 9. Comparison of number of pairs of patients with similar/different barcodes and number of similar clinical features: Plots showing the number of pairs of patients on the y axis and the number of similar clinical data on the x axis in TCGA dataset. Patients with similar PaSSSs and similar clinical data are shown in (**a**) for Peripheral blood samples (PB). Patients with similar PaSSSs and different clinical data are shown in (**b**) for Peripheral blood samples (PB). Patients with different PaSSSs and similar clinical data are shown in (**c**) for Peripheral blood samples (PB). The patients were grouped based on the following criteria for each clinical data – age : 21-40 years/ 41-60 years/60-80 years/ 80+ years. Gender: male/female. FLT3 mutation: present/absent. IDH1R132 mutation: present/absent. IDH1 R140 mutation: present/absent Hydroxyurea Treatment: Yes/No. PB blasts: >2/ <2 (where 2 is the fold values from healthy values). BM blasts: >2/ <2 (where 2 is the fold values from healthy values). Tumor stage: M0- M7.

Supplementary figure 10

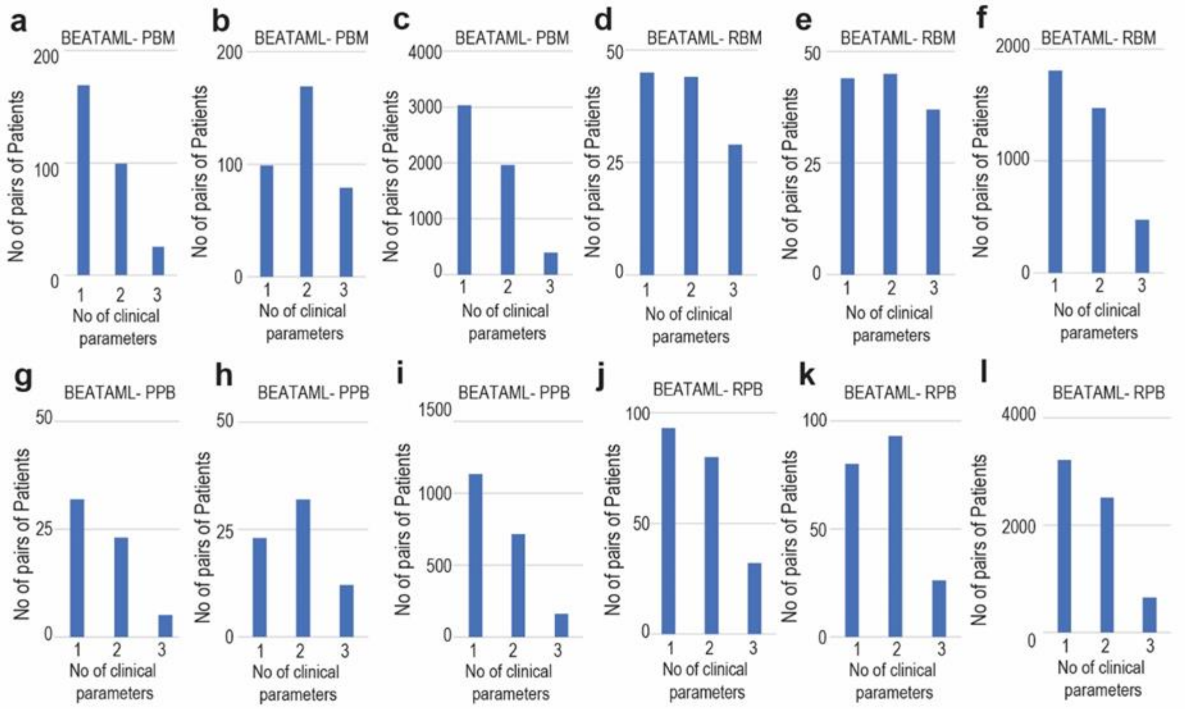

Supplementary figure 10. Comparison of number of pairs of patients with similar/different barcodes and number of similar clinical features. Plots showing the number of pairs of patients on the y axis and the number of similar clinical data on the x axis in BEATAML dataset. Patients with similar PaSSSs and similar clinical data are shown in (**a**), (**d**), (**g**) and (**j**) for Bone marrow (PBM & RBM) and Peripheral blood samples (PPB & RPB). Patients with similar PaSSSs and different clinical data are shown in (**b**), (**e**), (**h**) and (**k**) for Bone marrow (PBM & RBM) and Peripheral blood samples (PPB & RPB). Patients with different PaSSSs and similar clinical data are shown in (**c**), (**f**), (**i**) and (**l**) for Bone marrow (PBM & RBM) and Peripheral blood samples (PPB & RPB) The patients were grouped based on the following criteria for each clinical data – age: 0-20 years/21-40 years/ 41-60 years/60-80 years/ 80+ years. Gender: male/female. Tumor stage: adverse/favorable/ intermediate.

Supplementary figure 11

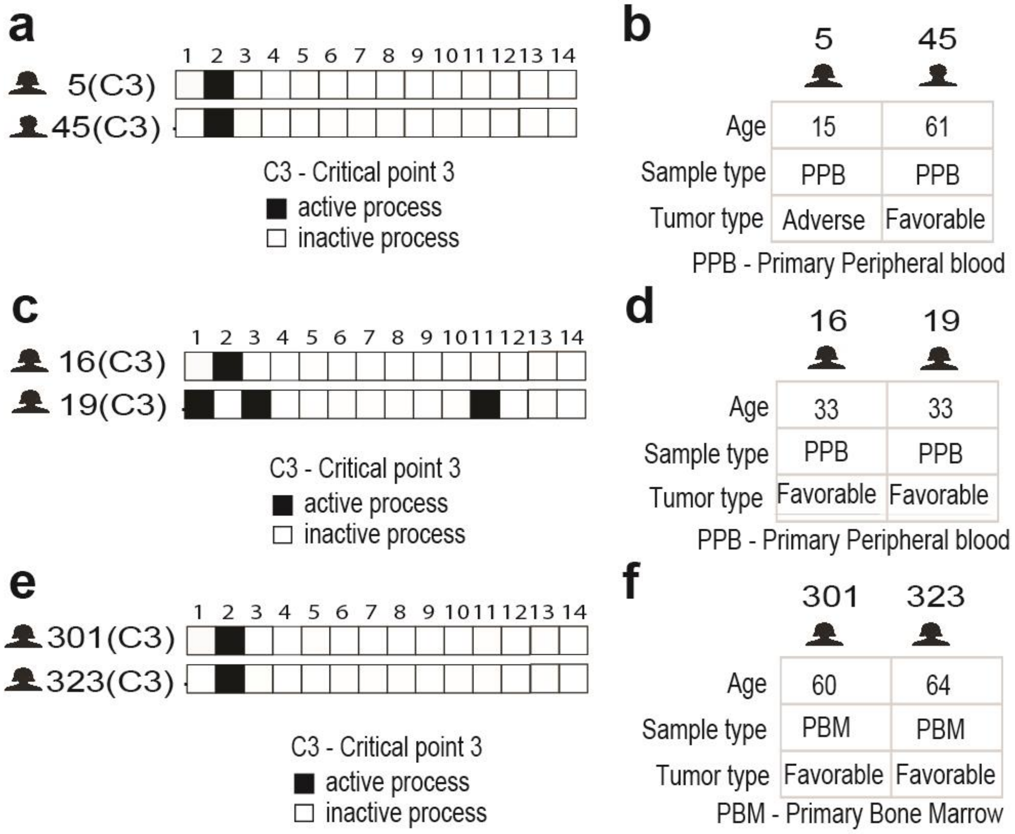

Supplementary figure 11. Patients with similar unbalanced processes can have different clinical data and patients with different barcodes can have the same clinical data. (**a**) Two patients (**BEATML**), having the same barcodes, are shown. (**b**) Clinical data of these two patients are shown, demonstrating that patients with same set of unbalanced processes can have different clinical data. (**c**) Two patients, having the different sets of unbalanced process are shown. (**d**) Clinical data of these two patients are shown, demonstrating that patients with different sets of unbalanced processes can have similar clinical data. (**e**) Two patients, having the same barcodes are shown. (**f**) Same clinical data of these two patients are shown, demonstrating that in some (rare) cases we find patients with same barcodes and the same clinical data.

Supplementary figure 12

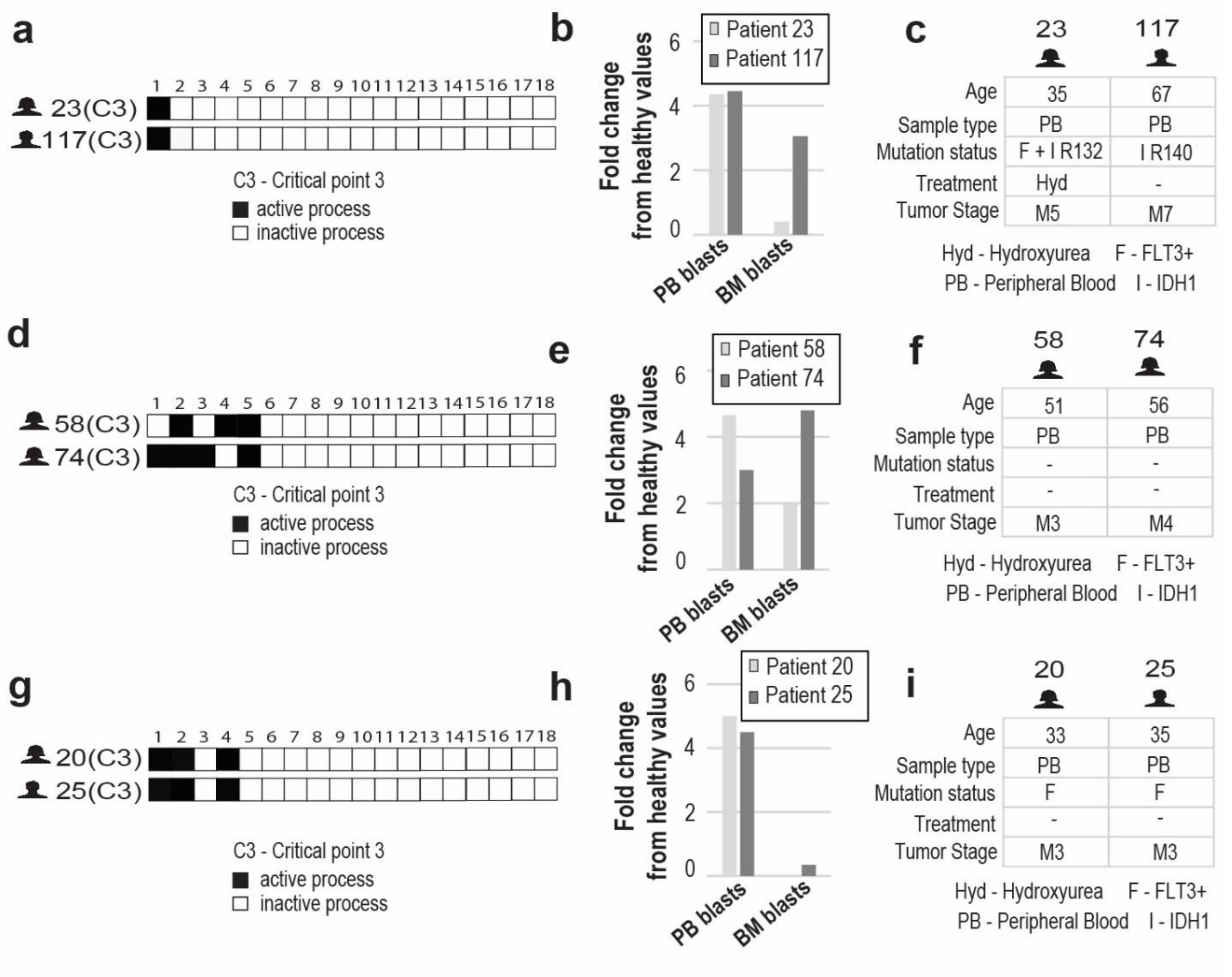

Supplementary figure 12. **Patients with similar unbalanced processes can have different clinical data and patients with different barcodes can have the same clinical data.** (**a**) Two patients (**TCGA**), having the same barcodes, are shown. (**b**) Fold change of different clinical markers are shown relative to their healthy values. (**c**) Different clinical data of these two patients are shown, demonstrating that patients with same set of unbalanced processes can have different clinical dat (BM blasts and panel c). (**d**) Two patients, having the different sets of unbalanced process are shown. (**e**) Fold change of different clinical markers are shown relative to their healthy values. (**f**) Clinical data of these two patients are shown, demonstrating that patients with different sets of unbalanced processes can have similar clinical data. (**g**) Two patients, having the same barcodes are shown. (**h**) Fold change of different clinical markers are shown relative to their healthy values. (**i**) Same clinical data of these two patients are shown, demonstrating that in some (rare) cases we find patients with same barcodes and the same clinical data.

Supplementary References

1. Nakase, K., Kita, K. & Katayama, N. IL-2/IL-3 interplay mediates growth of CD25 positive acute myeloid leukemia cells. *Med Hypotheses* **115**, 5–7 (2018).

2. Kaser, E. C. *et al.* The role of various interleukins in acute myeloid leukemia. *Medical Oncology* **38**, 55 (2032).

3. Cozzolino, F. *et al.* Interleukin 1 as an autocrine growth factor for acute myeloid leukemia cells. *Proc Natl Acad Sci U S A* **86**, 2369–2373 (1989).

4. Vijay, V. *et al.* Interleukin-8 blockade prevents activated endothelial cell mediated proliferation and chemoresistance of acute myeloid leukemia. *Leuk Res* **84**, 106180 (2019).

5. Nishioka, C., Ikezoe, T., Pan, B., Xu, K. & Yokoyama, A. MicroRNA-9 plays a role in interleukin-10-mediated expression of E-cadherin in acute myelogenous leukemia cells. *Cancer Sci* **108**, 685–695 (2017).

6. Porcu, P. *et al.* Hyperleukocytic leukemias and leukostasis: a review of pathophysiology, clinical presentation and management. *Leuk Lymphoma* **39**, 1–18 (2000).

7. Nourshargh, S. & Alon, R. Leukocyte migration into inflamed tissues. *Immunity* **41**, 694–707 (2014).

8. Engelman, J. A. Targeting PI3K signalling in cancer: opportunities, challenges and limitations. *Nature Reviews Cancer 2009 9:8* **9**, 550–562 (2009).

9. Yuan, T., Oncogene, L. C.- & 2008, undefined. PI3K pathway alterations in cancer: variations on a theme. *nature.com*.

10. Shlush, L. I. *et al.* Identification of pre-leukaemic haematopoietic stem cells in acute leukaemia. *Nature* **506**, 328–333 (2014).

11. Welch, J. S. *et al.* The origin and evolution of mutations in acute myeloid leukemia. *Cell* **150**, 264–278 (2012).

12. Rodrigues, A. C. B. da C. *et al.* Cell signaling pathways as molecular targets to eliminate AML stem cells. *Crit Rev Oncol Hematol* **160**, 103277 (2021).

13. Genomic and Epigenomic Landscapes of Adult De Novo Acute Myeloid Leukemia. *New England Journal of Medicine* **368**, 2059–2074 (2013).

14. Mondet, J., Chevalier, S. & Mossuz, P. Pathogenic Roles of S100A8 and S100A9 Proteins in Acute Myeloid and Lymphoid Leukemia: Clinical and Therapeutic Impacts. *Molecules* **26**, (2021).

15. Srikrishna, G. Fax +41 61 306 12 34 E-Mail karger@karger.ch S100A8 and S100A9: New Insights into Their Roles in Malignancy. *J Innate Immun* **4**, 31–40 (2012).

16. Goyette, J. & Geczy, C. L. Inflammation-associated S100 proteins: new mechanisms that regulate function. *Amino Acids 2010 41:4* **41**, 821–842 (2010).

17. Laouedj, M. *et al.* S100A9 induces differentiation of acute myeloid leukemia cells through TLR4. *Blood* **129**, 1980–1990 (2017).

18. Øyan, A. M. *et al.* CD34 expression in native human acute myelogenous leukemia blasts: Differences in CD34 membrane molecule expression are associated with different gene expression profiles. *Cytometry B Clin Cytom* **64B**, 18–27 (2005).

19. Zheng, J. *et al.* Integrative Analysis of Multi-Omics Identified the Prognostic Biomarkers in Acute Myelogenous Leukemia. *Front Oncol* **10**, 591937 (2020).

20. Ma, B., Simala-Grant, J. L. & Taylor, D. E. Fucosylation in prokaryotes and eukaryotes. *Glycobiology* **16**, (2006).

21. Cheng, L. *et al.* FUT family mediates the multidrug resistance of human hepatocellular carcinoma via the PI3K/Akt signaling pathway. *Cell Death Dis* **4**, e923 (2013).

22. Dai, Y. *et al.* Prognostic value of the FUT family in acute myeloid leukemia. *Cancer Gene Therapy 2019 27:1* **27**, 70–80 (2019).

23. Xiao, B. *et al.* Homer Regulates the Association of Group 1 Metabotropic Glutamate Receptors with Multivalent Complexes of Homer-Related, Synaptic Proteins. *Neuron* **21**, 707–716 (1998).

24. Ishiguro, K. & Xavier, R. Homer-3 regulates activation of serum response element in T cells via its EVH1 domain. *Blood* **103**, 2248–2256 (2004).

25. Shiraishi, Y., Mizutani, A., Yuasa, S., Mikoshiba, K. & Furuichi, T. Differential expression of Homer family proteins in the developing mouse brain. *Journal of Comparative Neurology* **473**, 582–599 (2004).

26. Brakeman, P. R. *et al.* Homer: a protein that selectively binds metabotropic glutamate receptors. *Nature 1997 386:6622* **386**, 284–288 (1997).

27. Stirewalt, D. L. *et al.* Identification of genes with abnormal expression changes in acute myeloid leukemia. *Genes Chromosomes Cancer* **47**, 8–20 (2008).

28. Moskow, J. J., Bullrich, F., Huebner, K., Daar, I. O. & Buchberg, A. M. Meis1, a PBX1-related homeobox gene involved in myeloid leukemia in BXH-2 mice. *Mol Cell Biol* **15**, 5434–5443 (1995).

29. Moens, C. B. & Selleri, L. Hox cofactors in vertebrate development. *Dev Biol* **291**, 193–206 (2006).

30. Liu, J. *et al.* Meis1 is critical to the maintenance of human acute myeloid leukemia cells independent of MLL rearrangements. *Ann Hematol* **96**, 567–574 (2017).

31. Fiúza, U. M. & Arias, A. M. Cell and molecular biology of Notch. *J Endocrinol* **194**, 459–474 (2007).

32. Bray, S. J. Notch signalling: a simple pathway becomes complex. *Nat Rev Mol Cell Biol* **7**, 678–689 (2006).

33. Czemerska, M. *et al.* Jagged-1: a new promising factor associated with favorable prognosis in patients with acute myeloid leukemia. *Leuk Lymphoma* **56**, 401–406 (2015).

34. Vaikari, V. P., Jang, M., Akhtari, M. & Alachkar, H. CD99 Is Highly Expressed in Acute Myeloid Leukemia (AML) and Presents a Viable Therapeutic Target. *Blood* **128**, 1540–1540 (2016).

35. Liu, W. *et al.* CDK6 Is a Potential Prognostic Biomarker in Acute Myeloid Leukemia. *Front Genet* **11**, 1771 (2021).

36. Gavet, O. *et al.* The stathmin phosphoprotein family: intracellular localization and effects on the microtubule network. *J Cell Sci* **111**, 3333–3346 (1998).

37. STMN1 promotes the proliferation and inhibits the apoptosis of acute myeloid leukemiacells by activating the PI3K/Akt pathway. https://www.techscience.com/biocell/v46n1/44767/html.

38. Jiang, W., Huang, S., Song, L. & Wang, Z. STMN1, a prognostic predictor of esophageal squamous cell carcinoma, is a marker of the activation of the PI3K pathway. *Oncol Rep* **39**, 834–842 (2018).

39. Nepstad, I., Hatfield, K. J., Grønningsæter, I. S. & Reikvam, H. The PI3K-Akt-mTOR Signaling Pathway in Human Acute Myeloid Leukemia (AML) Cells. *Int J Mol Sci* **21**, 2907 (2020).

40. Medina, K. L. Flt3 Signaling in B Lymphocyte Development and Humoral Immunity. *Int J Mol Sci* **23**, (2022).

41. Bonardi, F. *et al.* A Proteomics and Transcriptomics Approach to Identify Leukemic Stem Cell (LSC) Markers. *Molecular & Cellular Proteomics* **12**, 626–637 (2013).

42. Ozeki, K. *et al.* Biologic and clinical significance of the FLT3 transcript level in acute myeloid leukemia. *Blood* **103**, 1901–1908 (2004).

43. Birkenmeier, E. H. *et al.* Tissue-specific expression, developmental regulation, and genetic mapping of the gene encoding CCAAT/enhancer binding protein. *Genes Dev* **3**, 1146–1156 (1989).

44. Antonson, P. & Xanthopoulos, K. G. Molecular cloning, sequence, and expression patterns of the human gene encoding CCAAT/enhancer binding protein alpha (C/EBP alpha). *Biochem Biophys Res Commun* **215**, 106–113 (1995).

45. Landschulz, W. H., Johnson, P. F., Adashi, E. Y., Graves, B. J. & McKnight, S. L. Isolation of a recombinant copy of the gene encoding C/EBP. *Genes Dev* **2**, 786–800 (1988).

46. Wang, Q. F., Cleaves, R., Kummalue, T., Nerlov, C. & Friedman, A. D. Cell cycle inhibition mediated by the outer surface of the C/EBPalpha basic region is required but not sufficient for granulopoiesis. *Oncogene* **22**, 2548–2557 (2003).

47. Pabst, T. & Mueller, B. U. Complexity of CEBPA dysregulation in human acute myeloid leukemia. *Clinical Cancer Research* **15**, 5303–5307 (2009).

48. Preudhomme, C. *et al.* Favorable prognostic significance of CEBPA mutations in patients with de novo acute myeloid leukemia: a study from the Acute Leukemia French Association (ALFA). *Blood* **100**, 2717–2723 (2002).

49. Imazu, T. *et al. Bcl-2/E1B 19 kDa-Interacting Protein 3-like Protein (Bnip3L) Interacts with Bcl-2/Bcl-x L and Induces Apoptosis by Altering Mitochondrial Membrane Permeability*. http://www.stockton-press.co.uk/onc.

50. Handschuh, L. *et al.* Gene expression profiling of acute myeloid leukemia samples from adult patients with AML-M1 and -M2 through boutique microarrays, real-time PCR and droplet digital PCR. *Int J Oncol* **52**, 656 (2018).

51. Ye, J., Luo, D., Yu, J. & Zhu, S. Transcriptome analysis identifies key regulators and networks in Acute myeloid leukemia. *Acute myeloid leukemia* **24**, 487–491 (2019).

52. Dickson, G. J. *et al.* HOXA/PBX3 knockdown impairs growth and sensitizes cytogenetically normal acute myeloid leukemia cells to chemotherapy. *Haematologica* **98**, 1216 (2013).

53. Li, Z. *et al.* Up-regulation of a HOXA-PBX3 homeobox-gene signature following down-regulation of miR-181 is associated with adverse prognosis in patients with cytogenetically abnormal AML. *Blood* **119**, 2314–2324 (2012).

54. Vasudevan, S., Flashner-Abramson, E., Remacle, F., Levine, R. D. & Kravchenko-Balasha, N. Personalized disease signatures through information-theoretic compaction of big cancer data. *Proc Natl Acad Sci U S A* 201804214 (2018) doi:10.1073/pnas.1804214115.

55. Flashner-Abramson, E., Vasudevan, S., Adejumobi, I. A., Sonnenblick, A. & Kravchenko-Balasha, N. Decoding cancer heterogeneity: Studying patient-specific signaling signatures towards personalized cancer therapy. *Theranostics* **9**, (2019).
